# Multidecadal changes in functional diversity lag behind the recovery of taxonomic diversity

**DOI:** 10.1002/ece3.8381

**Published:** 2021-11-23

**Authors:** Nathan Jay Baker, Francesca Pilotto, Phillip Joschka Haubrock, Burkhard Beudert, Peter Haase

**Affiliations:** ^1^ Department of River Ecology and Conservation Senckenberg Research Institute and Natural History Museum Frankfurt Gelnhausen Germany; ^2^ Department of Historical, Philosophical and Religious Studies Environmental Archaeology Lab Umeå University Umeå Sweden; ^3^ Faculty of Fisheries and Protection of Waters South Bohemian Research Center of Aquaculture and Biodiversity of Hydrocenoses University of South Bohemia in České Budějovice Vodňany Czech Republic; ^4^ Department of Conservation and Research Bavarian Forest National Park Grafenau Germany; ^5^ Faculty of Biology University of Duisburg‐Essen Essen Germany

**Keywords:** freshwater, functional diversity, functional redundancy, long term, long‐term ecosystem research, macroinvertebrate, protected area

## Abstract

While there has been increasing interest in how taxonomic diversity is changing over time, less is known about how long‐term taxonomic changes may affect ecosystem functioning and resilience. Exploring long‐term patterns of functional diversity can provide key insights into the capacity of a community to carry out ecological processes and the redundancy of species’ roles. We focus on a protected freshwater system located in a national park in southeast Germany. We use a high‐resolution benthic macroinvertebrate dataset spanning 32 years (1983–2014) and test whether changes in functional diversity are reflected in taxonomic diversity using a multidimensional trait‐based approach and regression analyses. Specifically, we asked: (i) How has functional diversity changed over time? (ii) How functionally distinct are the community's taxa? (iii) Are changes in functional diversity concurrent with taxonomic diversity? And (iv) what is the extent of community functional redundancy? Resultant from acidification mitigation, macroinvertebrate taxonomic diversity increased over the study period. Recovery of functional diversity was less pronounced, lagging behind responses of taxonomic diversity. Over multidecadal timescales, the macroinvertebrate community has become more homogenous with a high degree of functional redundancy, despite being isolated from direct anthropogenic activity. While taxonomic diversity increased over time, functional diversity has yet to catch up. These results demonstrate that anthropogenic pressures can remain a threat to biotic communities even in protected areas. The differences in taxonomic and functional recovery processes highlight the need to incorporate functional traits in assessments of biodiversity responses to global change.

## Graphical abstract

Recent biodiversity time‐series analyses have mainly focused on taxonomic diversity; however, there is much to learn about how the recovery of taxonomy may lead to shifts in ecosystem functioning and resilience, particularly in the long term. Focusing on a protected stream benthic macroinvertebrate community, we investigated and compared patterns of functional and taxonomic diversity over a 32‐year period. Besides identifying non‐linear trends and differences among functional and taxonomic diversity, we show that the recovery of functional diversity lags behind that of taxonomic diversity. Accordingly, we demonstrate that reported taxonomic changes are not always straightforward and could even be complex and/or contradictory and that by incorporating functional diversity measures into analyses, we can gain a more holistic picture regarding biodiversity responses in the Anthropocene.

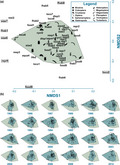

## 1 INTRODUCTION

In the Anthropocene, changes to global biodiversity are widespread and dynamic, albeit heterogeneous across spatial scales and taxonomic groups (Dornelas et al., 2014; Pilotto et al., 2020). At local scales, temporal patterns of biodiversity responses are highly variable, with declines (Habel et al., 2016; Hallmann et al., 2017; Thomas, 2016), increases (e.g., Haase et al., 2019), and mixed results (Baranov et al., 2020) all commonly reported. Consequently, long‐term monitoring of biodiversity change at local scales is critical for conservation planning (Jarzyna & Jetz, 2018).

Even when reliable and representative long‐term data are available, investigating biodiversity patterns is not without its challenges, particularly as local biodiversity is indisputably governed by biotic and abiotic interactions and comprises a multitude of different facets, namely taxonomic diversity (TD), phylogenetic diversity (PD), and functional diversity (FD), all of which are important for maintaining ecosystem functioning (Devictor et al., 2010). Two commonly investigated facets of biodiversity include TD and FD, whereby TD is defined as the richness and evenness (i.e., abundance distributions) of organisms in a given ecosystem and FD as “*the value and range of functional traits of the organisms in a given ecosystem*” (Tilman, 2001). Recently, the investigation of FD in biodiversity research has grown in popularity, with multivariate trait‐based approaches becoming one of the most widely used measures of FD (Schmera et al., 2017).

Spatial and temporal dynamics in FD result from various interwoven assembly mechanisms, such as biotic interactions, niche dynamics, and environmental filtering (Múrria et al., 2020). Thus, investigating FD as a measure of biodiversity can provide orthogonal yet complementary information to that of TD as it has been found to be more appropriate for (i) elucidating the link between ecosystem functionality and biodiversity, (ii) providing a more mechanistic understanding of biodiversity−environment relationships, and (iii) setting a baseline for which biodiversity patterns can be compared across various spatial scales and between communities composed of different species (Múrria et al., 2020; Pilière et al., 2016; Verberk et al., 2013).

One of the driving forces behind the initiation of long‐term ecosystem monitoring programs in Europe was water acidification, primarily due to sulfur (SO_2_) deposition in the 20th century (Beudert & Gietl, 2015; Garmo et al., 2014). Surface waters, particularly at higher latitudes and altitudes (i.e., headwaters), were severely impacted by SO_2_ deposition, and the resulting acidification threatened both the quality of drinking water and many sensitive aquatic freshwater fauna, homogenizing the community to contain mostly resistant taxa (Alewell et al., 2001). Acidification resulted in changes in the taxonomic and functional composition of benthic macroinvertebrate communities. Acidified streams often had low species richness and were dominated by acid‐tolerant taxa (e.g., Plecoptera), with a loss of acid‐sensitive taxa (e.g., several Ephemeroptera species). Communities were dominated by shredders and predators, while scrapers became rare or absent (Baldigo et al., 2009; Traister et al., 2013). Since the 1980s, however, increased regulation of acidifying pollutants has led to improved conditions of Europe's freshwater environments (Stoddard et al., 1999; Velle et al., 2016).

Ecological recovery from acidification and other anthropogenic impacts would manifest as improvements not only in TD but also in FD (Van Looy et al., 2019). Recent studies have found positive long‐term trends in both TD and FD of freshwater invertebrate communities (Bruno et al., 2019; Floury et al., 2018; Mouton et al., 2020). However, warming water temperatures are also facilitating the migration of thermophilic taxa into previously uninhabitable environments (Mouton et al., 2020). These processes have resulted in compositional restructuring (Larsen et al., 2018) with specialist taxa either lost or restricted to reduced habitats (Bruno et al., 2019) and leading to increased biotic homogenization (Floury et al., 2018; Mouton et al., 2020). However, even when functional recovery is facilitated by increased taxonomic diversity, previously extirpated organisms with suitable trait combinations may not be able to recolonize due to dispersal limitations, a lack of appropriate colonizers in the meta‐community, and/or competition (Traister et al., 2013).

Our study was conducted at a protected headwater system, the Grosse Ohe River, located within a national park in southeast Germany. The system has experienced considerable changes in TD between 1983 and 2014; benthic macroinvertebrate abundance increased by 173% and richness by 51.6% resulting in taxonomic restructuring driven by a disproportional increase in Diptera (Baker et al., 2021). Major stressors to the Grosse Ohe River ecosystem include historic acidification, bark beetle‐induced spikes in nitrate concentrations, and climate warming. Before c.a. 2000, a decline in sulfur deposition and subsequent acidification recovery led to increases in abundance and taxonomic richness. After the turn of the 20th century, bark beetle outbreak‐induced increases in nitrogen concentrations coupled with warming temperatures (+1.5°C) likely drove the taxonomic restructuring and dominance of the Diptera at the expense of taxa such as the Plecoptera and Trichoptera (Baker et al., 2021).

Here, we investigate whether those changes in TD over multidecadal timescales translate into complementary changes in the FD. When changes in FD and TD are paralleled, low functional redundancy is expected. In contrast, high TD but low FD suggests a functionally homogenous community (Micheli & Halpern, 2005). Thus, in this study, we asked the following:

1. How has the FD (see Table 1) of the Grosse Ohe benthic macroinvertebrate communities changed through time? To what extent has the occupied niche space shifted over time? Previous work at this site found a recovery of TD over time (Baker et al., 2021). We hypothesize FD additionally will increase over time (*H*_1a_), enlarging the occupied niche space (*H*_1b_).
2. How functionally distinct are the taxa within the Grosse Ohe macroinvertebrate communities? A higher occurrence of specialized taxa is expected considering the dendritic and disconnected nature of headwater stream networks (Heino, 2005). We hypothesize that, given the biological recovery that followed extensive acidification, the different taxonomic groups present within the Grosse Ohe River ecosystem are highly functionally distinct and that the overall functional distinctiveness will increase analogous to functional diversity (*H*_2_).
3. Are changes in TD concurrent with changes in FD? When ecosystems are degraded, functionally distinct taxa may be lost (but see Mouton et al., 2020). Contrastingly, when ecosystems recover, FD may have a lagged or asymmetric increase following the recovery of TD (Micheli & Halpern, 2005). We hypothesize parallel increases in TD and FD (*H*_3a_). However, we expect that the changes in FD compared with TD will be less pronounced (*H*_3b_).
4. What is the extent of functional redundancy within the Grosse Ohe macroinvertebrate communities? Owing to the linear increases in TD reported in Baker et al. (2021) and our expectations that FD would concurrently increase over time, we hypothesize a low degree of functional redundancy within the macroinvertebrate communities (*H*_4_) (Micheli & Halpern, 2005).

**TABLE 1:** Conceptual and mathematical definitions of all metrics and measurements used in this study

Metric	Conceptual definition	Mathematical definition
Taxonomic richness	The number of taxonomically distinct species within a community	The summation of all taxonomically distinct species within a given community
Taxonomic evenness	The distribution of abundances across all species within a community	The regularity with which species abundances are distributed across the various contributing species in a given community
Taxonomic turnover	The loss and/or gain of species in a community over time (i.e., species replacement between communities)^a^	The percentage of dissimilarity in species composition (alpha diversity) between two communities^a^
Functional turnover	The loss and/or gain of unique functional traits over time (i.e., differences in functional strategies between communities)^a^	The percentage of dissimilarity in functional trait membership states (CWM) between two communities^a^
Community‐weighted mean (CWM)	Abundance‐weighted trait membership state proportions. An index of functional composition^b^.	The mean trait value of species weighted by the species abundances^c^. Used to compute the multidimensional trait space^b^
Functional richness (FRic)	The amount of niche space occupied by all species within a given community^c^	The convex hull volume (i.e., the smallest polygon) of the individual species in multidimensional trait space for a given community^d^, ^e^
Functional evenness (FEve)	The distribution of abundances across the niche space (i.e., in each trait)^d^. Like the “classical” evenness metric but based on trait information	The regularity with which species abundances are distributed along the minimum spanning tree, which links all the species in the multidimensional functional space^d^, ^e^
Functional divergence (FDiv)	The degree to which the abundance distribution utilizes [maximizes] differences in traits within the community^e^, ^f^. In order words, a measure of how spread or clumped species are within the niche space, weighted by the relative abundance^c^, ^g^	The species deviance from the mean distance to the center of gravity weighted by relative abundance within multidimensional trait space^d^, ^e^
Functional dispersion (FDis)	The average distance of individual species to the group centroids of all species^h^	The weighted (i.e., species relative abundances) mean distance in multidimensional trait space of individual species to the centroid of all species^d^, ^h^
Rao's quadratic entropy (RaoQ)	The functional differences between two randomly selected species in the niche space^i^, ^j^	The sum of the pairwise distances between species in multidimensional trait space weighted by their relative abundance^d^, ^j^
Overarching convex hull	Total amount of available trait space (i.e., the overall niche space occupied by all species from all years combined)^k^	The convex hull volume (i.e., the smallest polygon) of the individual species in multidimensional trait space for all communities combined^d^, ^e^
Functional distinctiveness (FDist)	The degree to which species are functionally dissimilar from other species in a given community^l^	The weighted (i.e., species relative abundances) functional distance from an individual species to all other species in the given community^l^
Functional redundancy (FRed)	The relative amount of taxonomically distinct species that exhibit similar ecological functions^m^. In other words, a situation whereby species share similar functions in the community^n^	The degree to which species can be exchanged within a community without the loss of ecological functionality^n^

^a^ Villéger et al. (2013).

^b^ Laliberté et al. (2014).

^c^ Mason et al. (2005).

^d^ Pakeman (2014).

^e^ Villéger et al. (2008).

^f^ Laliberté and Legendre (2010).

^g^ Mouillot et al. (2014).

^h^ Legras et al. (2020).

^i^ Botta‐Dukát (2005).

^j^ Ricotta (2005).

^k^ Mammola and Cardosa (2020).

^l^ Violle et al. (2017).

^m^ Micheli and Halpern (2005).

^n^ Rosenfeld (2002).

## 2 MATERIALS AND METHODS

### 2.1 Site description

The Grosse Ohe River is a protected, low‐order mountainous stream in the strict conservation zone of the Bavarian Forest National Park (BFNP) in southeast Germany. It has its origins on the peaks of Mt. Grosser Rachel (altitude: 1453 m a.s.l.) and Mt. Plattenhausenriegel (altitude: 1372 m a.s.l.) and drains a catchment area of 19.1 km^2^ forming a dendritic network of heterogeneous low‐order headwater streams. Two of its tributaries drain the subcatchments of Seebach (13.3 km^2^ = 69%) and Vorderer Schachtenbach (5.9 km^2^ = 31%). Due to high relief energy and current velocities, the substrate comprises gravel, cobbles, and rocks. The catchment area is 98% forested with ~30% European beech *Fagus sylvatica* L. and ~60% Norway spruce *Picea abies* (L.), with the latter dominating the riparian vegetation. Within the BFNP, which encloses an area of 241 km^2^, the Grosse Ohe River represents one of 11 similar stream networks whose catchments have the same physiographic characteristics (bedrock, soils, and vegetation). All sampling was conducted downstream of the Taferlruck measuring station (48°56’17.99”N, 13°24’45.13”E), a permanent monitoring locality of the German Long‐Term Ecosystem Research (LTER) network (Haase et al., 2016; Mirtl et al., 2018) and the International UNECE ICP Waters networks (Kvaeven et al., 2001; Velle et al., 2016). The distance from the sampled stream section to non‐protected landscape elements and settlements is at least 3.5 km. See Baker et al. (2021) for further details.

### 2.2 Macroinvertebrate communities

Long‐term and high‐resolution sampling of the Grosse Ohe River benthic macroinvertebrate communities has been discontinuously conducted over a 32‐year period from 1983 to 2014 (24 annual sampling surveys representing 78% of the total period covered). Records include 170 taxa mostly identified to genus and species level (Baker et al., 2021). To ensure homogeneity and consistency, only spring and summer sampling surveys were included in analyses. Sampling was conducted using multihabitat kick sampling throughout the study. Prior to 2004, abundance estimates were assigned to predetermined abundance classes (Braukmann, 2000), whereas after 2004, true abundance (individuals per square meter) data were recorded (Haase et al., 2004). For consistency, the true abundances of the taxa recorded after 2004 were standardized to abundance classes. The standardized abundance classes were then converted to the mean abundance of each abundance class (see Baker et al., 2021). Despite these necessary adjustments, there was no significant impact of the count method on the analyzed community metrics, which included compositional and abundance metrics, richness and diversity metrics, tolerance and sensitivity metrics, and functional feeding group proportions (Baker et al., 2021).

### 2.3 Taxonomic resolution

Most macroinvertebrate trait data are available at the genus level (Schmera et al., 2017), and we used genus‐level data in the absence of species‐level trait information. Additionally, we moved dipteran taxa to the subfamily or tribe level when finer resolution trait information was missing. For the Coleoptera, no delineation between adult and larval stages was made. This reduced the number of taxa from 170 to 70 taxa complexes but still included all individuals. Using this adjusted taxon list, we constructed a multidimensional trait space and calculated functional diversity metrics. As a quality control measure, we used the adjusted taxa list to calculate measures of TD, analyzed their trends via linear trend analyses (*mmkh* function in the modifiedmk R package; Patakamuri & O’Brien, 2020), and compared the results with those presented in Baker et al. (2021). Further, to investigate whether temporal patterns of taxonomic diversity were robust to changes in taxonomic resolution, we calculated taxonomic richness, evenness, and temporal turnover on both the original (170 taxa) and adjusted (70 taxa) taxa lists. Spearman's rank correlation was used to evaluate the correlation between the trends of the two taxa lists. Temporal taxonomic patterns in the original and adjusted taxon lists were correlated: taxonomic richness (*R* ^2^ = 0.81, *p* < .001), taxonomic evenness (*R* ^2^ = 0.94, *p* < .001), and taxonomic turnover (*R* ^2^ = .74, *p* < .001). See Appendix S1 for more detailed results of the quality control measures.

To ensure consistency and comparability of our taxa lists with contemporary taxonomic nomenclature, the adjusted taxa list was harmonized according to the operational taxa list of German macroinvertebrates (Haase et al., 2006) and taxa names were validated using the *Taxa Validation Tool* on the website www.freshwaterecology.info (Schmidt‐Kloiber & Hering, 2015).

### 2.4 Functional traits

Following Schmera et al. (2017), we only used biological traits (i.e., traits pertaining to the biological, morphological, and life history of an animal in relation to their reproduction, growth, and survival; Violle et al., 2017) to describe the benthic macroinvertebrate community functional diversity. Eleven trait groups with 63 traits (i.e., modalities) were included in our calculations (Appendix S2). Trait data were primarily sourced from Tachet et al. (2010), but when missing, trait information was supplemented from the AQEM/STAR trait database (AQEM consortium, 2004), www.freshwaterecology.info (Schmidt‐Kloiber & Hering, 2015), and the DISPERSE database (Sarremejane et al., 2020). All trait information was fuzzy coded according to Chevenet et al. (1994) and proportionally scaled between 0 and 1 (Schmera et al., 2015).

### 2.5 Data analysis

First, using the convex hull method (Villéger et al., 2008), we investigated the change in FD over the observation period through distance‐based metrics, namely functional richness (FRic), functional evenness (FEve), functional divergence (FDiv), functional dispersion (FDis), Rao's quadratic entropy (RaoQ), and functional distinctiveness (FDist; see Table 1 for metric‐specific definitions). Despite FDis and RaoQ being highly correlated (*R* ^2^ = 0.96), we opted to include both as they are commonly used in freshwater macroinvertebrate research to increase comparability (Schmera et al., 2017). Following Mason et al. (2005), Mouchet et al. (2010), and Pavoine and Bonsall (2011), the selected functional metrics describe a community's niche space and encompass the three dimensions of FD, namely richness, regularity/evenness, and divergence (Table 1).

While FDis (Laliberté & Legendre, 2010) and RaoQ (Botta‐Dukát, 2005) are, by design, uncorrelated to taxonomic richness (Swenson, 2014), the other studied measures of FD, particularly FRic and FEve, are known to be strongly positively correlated to taxonomic richness (Gotelli & Graves, 1996). Thus, to ensure that the trends of FRic, FEve, and FDiv were independent from the trends in taxonomic richness, we used a “name shuffling” null model approach to compute standardized effect sizes (SES) of the functional metrics that correct for differences in taxonomic richness across samples (Swenson, 2014). We then compared the observed functional metric values with the S.E.S. functional metric values using a Pearson rank correlation, which revealed strong positive correlations (FRic *R* ^2^ = 0.83, *p* < .001; FEve *R* ^2^ = .57, *p* = .004; FDiv *R* ^2^ = 0.8, *p* < .001). Thus, to limit metric redundancy, we chose to only present the results of the observed functional metrics but provide more details pertaining to our null model approach in Appendix S3.

To calculate the distance‐based FD metrics, we constructed a taxon–trait matrix from which a Gower dissimilarity matrix was derived (*gowdis* function in the FD package; Laliberté et al., 2014). Functional diversity metrics for each sampling year were computed using the *dbFD* function in the FD package (Laliberté et al., 2014). We restricted the number of dimensions (m) to 13, which represented a good trade‐off between computation time and the quality of the reduced trait space, constrained FRic between 0 and 1 by standardizing FRic scores for each year by the total FRic containing all species (stand.FRic = TRUE), and scaled RaoQ by the highest value across all frequency distributions (scale. RaoQ = TRUE). To determine how the FD metrics changed over the observation period, each metric was assessed through linear trend analyses (*mmkh* function) and through non‐linear regression analyses (generalized additive modeling—*gam* function in the mgcv package; Wood, 2021). Prior to regression analyses, we normalized the distribution of FRic using base 10 log transformation. No linear or additive model residuals suggested a violation of the assumptions of normality and independence (Zuur et al., 2009).

We visualized the change in occupied niche space over the observation period using non‐metric multidimensional scaling (NMDS). It is important to note that we used NMDS simply as a visualization tool and that the computation of FD metrics was based on the multidimensional trait‐based approach detailed above (Botta‐Dukát, 2005; Laliberté & Legendre, 2010; Villéger et al., 2008). NMDS was conducted using the *metaNMDS* function in the vegan package (Oksanen et al., 2017) and the same Gower dissimilarity matrix. Using the overall niche space occupied by all taxa from all years as a reference (i.e., overarching convex hull; Table 1), we plotted the occupied niche space of the community in each year (i.e., annual convex hull), weighting each point (taxon) by its abundance in that year (Múrria et al., 2020).

Second, to describe the functional differences between the taxa present in the Grosse Ohe River, we calculated the functional distinctiveness (FDist) of the community over time. Using the Gower dissimilarity matrix, we calculated abundance‐weighted FDist using the *distinctiveness* function in the funrar package (Grenié et al., 2017). We first determined the change in FDist over the observation period using the annual abundance‐weighted mean FDist scores and then assessed temporal change using linear trend analyses and generalized additive modeling. To better understand the distribution and rarity of traits within the community, we additionally calculated FDist for each of the major taxonomic groups (i.e., Coleoptera, Diptera, Ephemeroptera, Oligochaeta, Plecoptera, and Trichoptera; Baker et al., 2021). We used an analysis of variance (ANOVA) with Tukey's post hoc test to determine differences in FDist between taxonomic groups.

Third, to determine whether changes in TD were intrinsically linked to FD, we calculated taxonomic richness, evenness, and turnover and compared these metrics with FRic, FEve, and functional turnover. For the calculation of taxonomic richness and evenness, we used the software ASTERICS, v4.04 (AQEM Consortium, 2013). To calculate annual taxonomic and functional turnover between pairs of consecutive years, we used the *turnover* function in the codyn package (Hallett et al., 2016). For functional turnover calculations, traits were used as “species” and abundance‐weighted trait membership state proportions (CWM; Table 1) were used as “abundances.” Temporal trends in the taxonomic and functional metrics were assessed through linear and non‐linear regression analyses. Spearman's rank correlation was used to evaluate correlations between the metric trends, and paired t tests were used to evaluate changes in mean taxonomic and functional temporal turnover.

Lastly, to determine the amount of functional redundancy (FRed) within the communities, we examined the correlation between TD and FD. The inclusion of a large number of traits can lead to a biased number of “functionally unique taxa” (i.e., high FD and low FRed; Legras et al., 2020). Therefore, we examined FRed by assessing the slope of the relationship between TD and FD (Micheli & Halpern, 2005) using taxonomic richness (i.e., the number of taxa) and FD metrics computationally independent from taxonomic richness (FDis and RaoQ).

Unless otherwise specified, all statistical analyses were conducted in the R statistical environment, version 3.6.3 (R Core Team, 2020).

## 3 RESULTS

### 3.1 Functional diversity metrics and niche space

**FIGURE 1:**
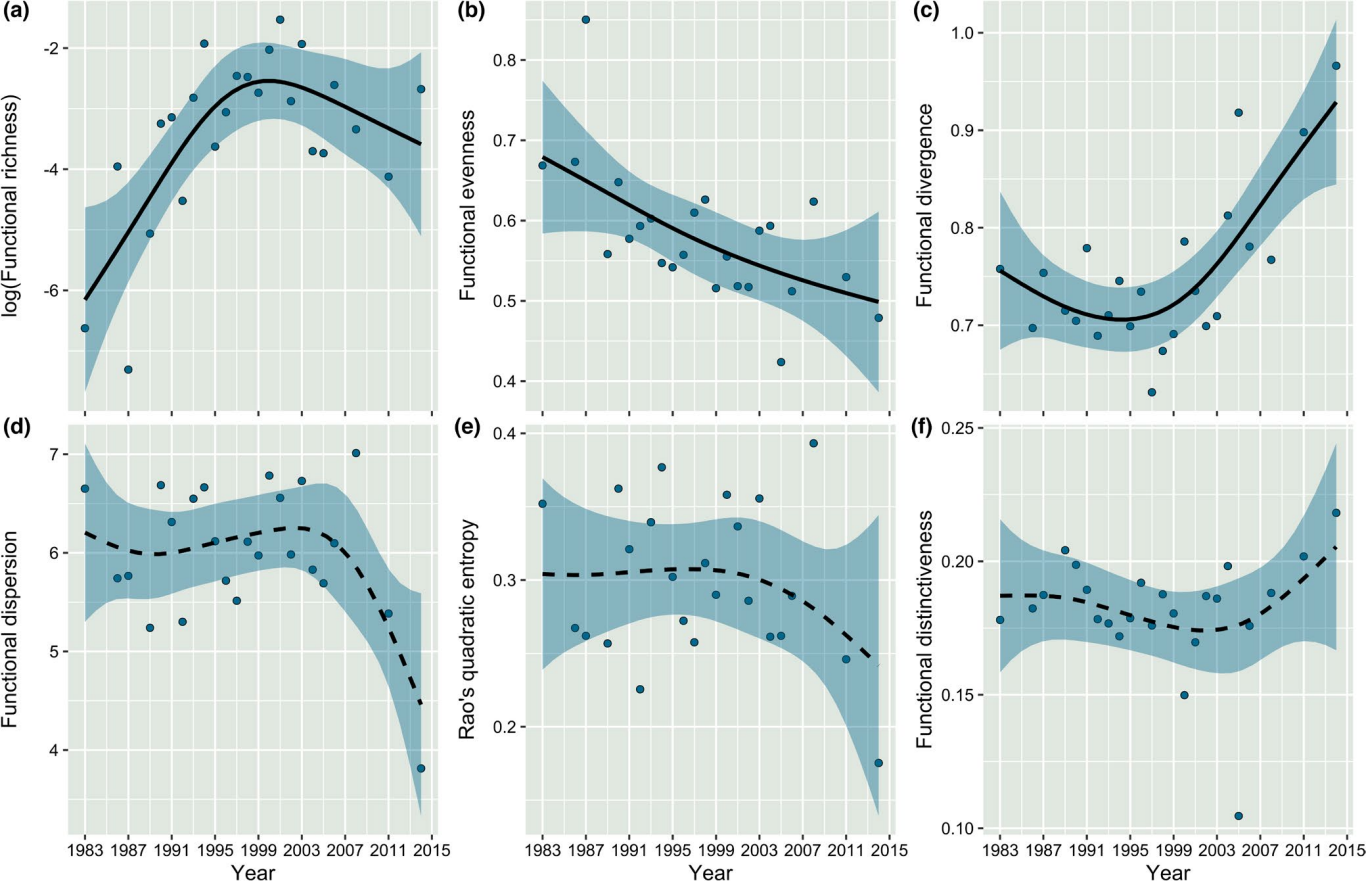
Generalized additive models (gam) exploring the relationship between the functional diversity metrics through time (year). The number of knots has been arbitrarily set at 6. Solid lines indicate significant change over time, whereas dashed lines represent no significant change. (a) log(Functional richness). (b) Functional evenness. (c) Functional divergence. (d) Functional dispersion. (e) Rao's quadratic entropy. (f) Functional distinctiveness

Over the three decades of observation, FEve decreased while FDiv increased (Table 2). This was evident using both Spearman's and Kendall's tau rank correlations (FEve: Spearman: *R* ^2^ = 0.63, Kendall: tau [T] = −0.46; FDiv: Spearman: *R* ^2^ = 0.46, Kendall: *T* = 0.3), and non‐linear regression analysis (FEve adj. *R* ^2^ = 0.38; FDiv adj. *R* ^2^ = 0.6; Figure 1b, c). FRic additionally had a significant non‐linear trend, increasing linearly up until the turn of the century but thereafter decreasing (FRic adj. *R* ^2^ = .55; Figure 1a). Neither FDis nor RaoQ (Figure 1d, e), both composite measures of FD, and FDist (Figure 1f) showed any significant trends over the observation period. The quality of the reduced trait space was 0.75 (see Appendix S4).

**TABLE 2:** Spearman's rank and Kendall's tau rank correlations of the change in functional diversity metrics over time

Metric	Spearman's rank correlation—rho			Kendall's rank correlation—tau		
	*S*	rho	*p*‐value	*T*	tau	*p*‐value
FRic	1398	0.392	.059	177	0.283	.055
FEve	**3754**	**−0.632**	.001 ***	**75**	**−0.457**	**<.001** ***
FDiv	**1242**	**0.460**	.025 *	**180**	**0.304**	.039*
FDis	2418	−0.051	.812	131	−0.051	.750
RaoQ	2608	−0.134	.531	123	−0.109	.476
FDist	2218	0.036	.869	141	0.022	.902

Note

*p*‐values in bold represent a significant change in a metric over time (*p* ≤ .05); *p < .05, **p < .01, ***p < .001.

Abbreviations: S, T, value of the test statistic, rho, tau, estimated measure of association.

**FIGURE 2:**
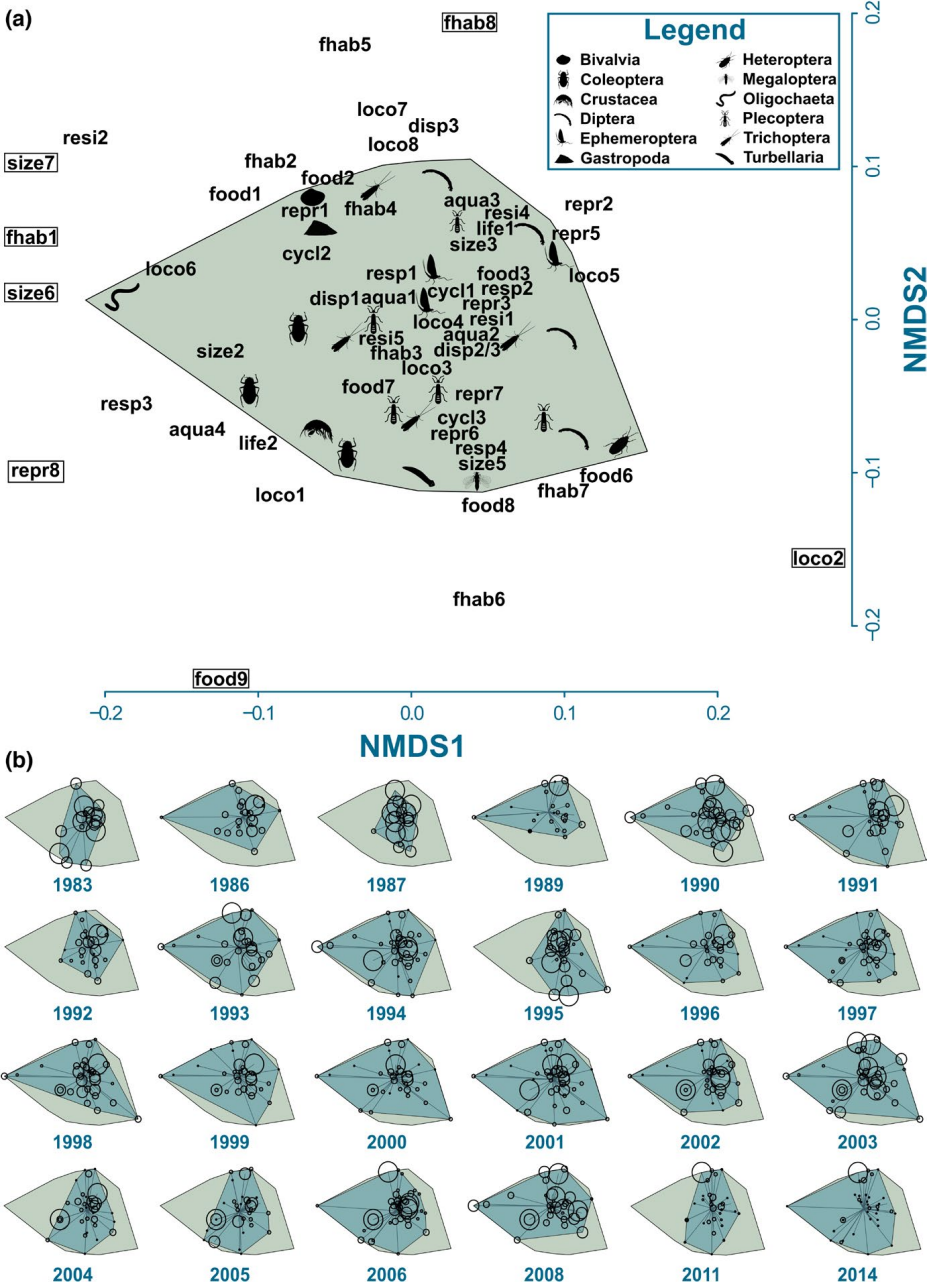
Non‐metric multidimensional scale (NMDS) plots to visualize the occupied niche space of the Grosse Ohe macroinvertebrate communities. (a) NMDS showing the overarching convex hull [and distribution of taxa within the niche space] of the communities from all years combined. Traits enclosed in boxes are outside the axis ranges of the presented NMDS. (b) NMDS plots showing the annual change in occupied niche space (annual convex hull) of the communities over time (in chronological order). In each plot, individual taxa are represented by a point and the circumferences of the points are scaled according to the most abundant taxon of that year. All silhouettes sourced from http://phylopic.org/ under a Creative Commons license. Please refer to Appendix S2 for trait abbreviations

The overarching convex hull, which indicates differences between taxonomic groups within the entire functional space, contained a dense clustering of traits and taxonomic groups in its center (Figure 2a) suggesting trait redundancy of the Ephemeroptera, Plecoptera, and Trichoptera. The Diptera spanned a large proportion of the niche space, suggesting more functionally distinct taxa. Coleoptera and Oligochaeta both had high functional distinctiveness relative to other taxa. The remaining taxonomic groups (Bivalvia, Crustacea, Gastropoda, Heteroptera, Megaloptera, and Turbellaria) not only form a small component of the community but also have relatively high degrees of functional distinctiveness.

The occupied niche space for each sampling year (FRic; Figure 2b) changed significantly and non‐linearly over the observation period. The proportion of occupied niche space increased linearly until ~2000, then declined (Figure 1a). The significant non‐linear changes in occupied niche space were accompanied by changes in the relative abundance of taxa distributed within the annual niche spaces (i.e., a decline in FEve; Figure 1b), highlighted by an increased dominance of the Diptera, particularly in 2011 and 2014 (Figure 2b; figure 4 in Baker et al., 2021). Moreover, the communities became increasingly more divergent throughout the observation period, highlighted by significant, non‐linear increases in FDiv (Figure 1c).

### 3.2 Functional distinctiveness

**FIGURE 3:**
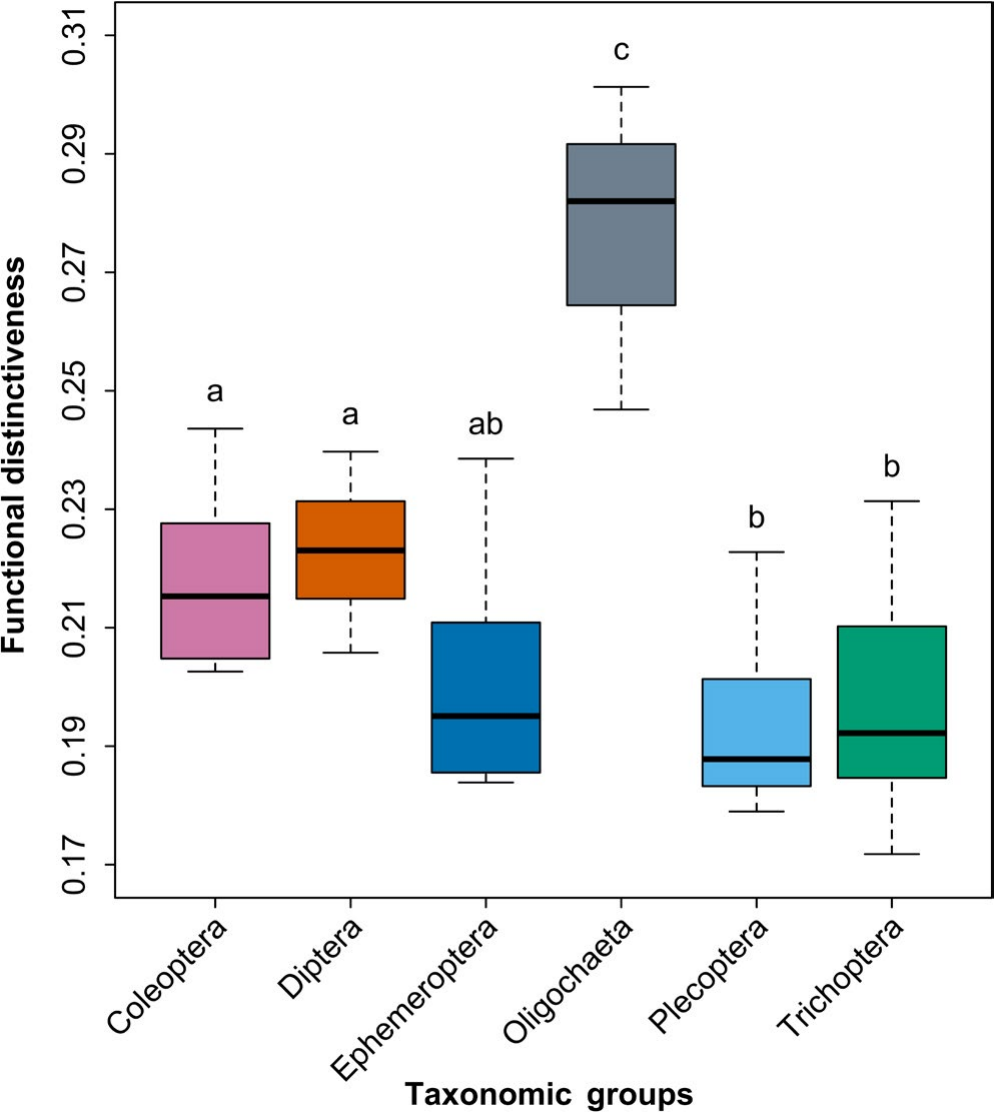
Boxplot indicating the functional distinctiveness (FDist) between taxonomic groups. Differing superscript letters denote significant differences between taxonomic groups. Colors of taxonomic groups align with those used in Figure 4 of Baker et al. (2021)

Taxonomic groups had significantly different FDist (Figure 3), confirming what was observed regarding the distribution of taxa within the niche space (Figure 2a). Oligochaeta were the most functionally distinct group (mean *F*Dist = 0.282; Figure 3). Coleoptera (mean *F*Dist = 0.215) and Diptera (mean *F*Dist = 0.223) showed significantly higher *F*Dist than Plecoptera (mean *F*Dist = 0.188) and Trichoptera (mean *F*Dist = 0.192). Ephemeroptera (mean *F*Dist = 0.195) showed no significant differences to any groups besides Oligochaeta.

### 3.3 Taxonomic vs functional diversity

**FIGURE 4:**
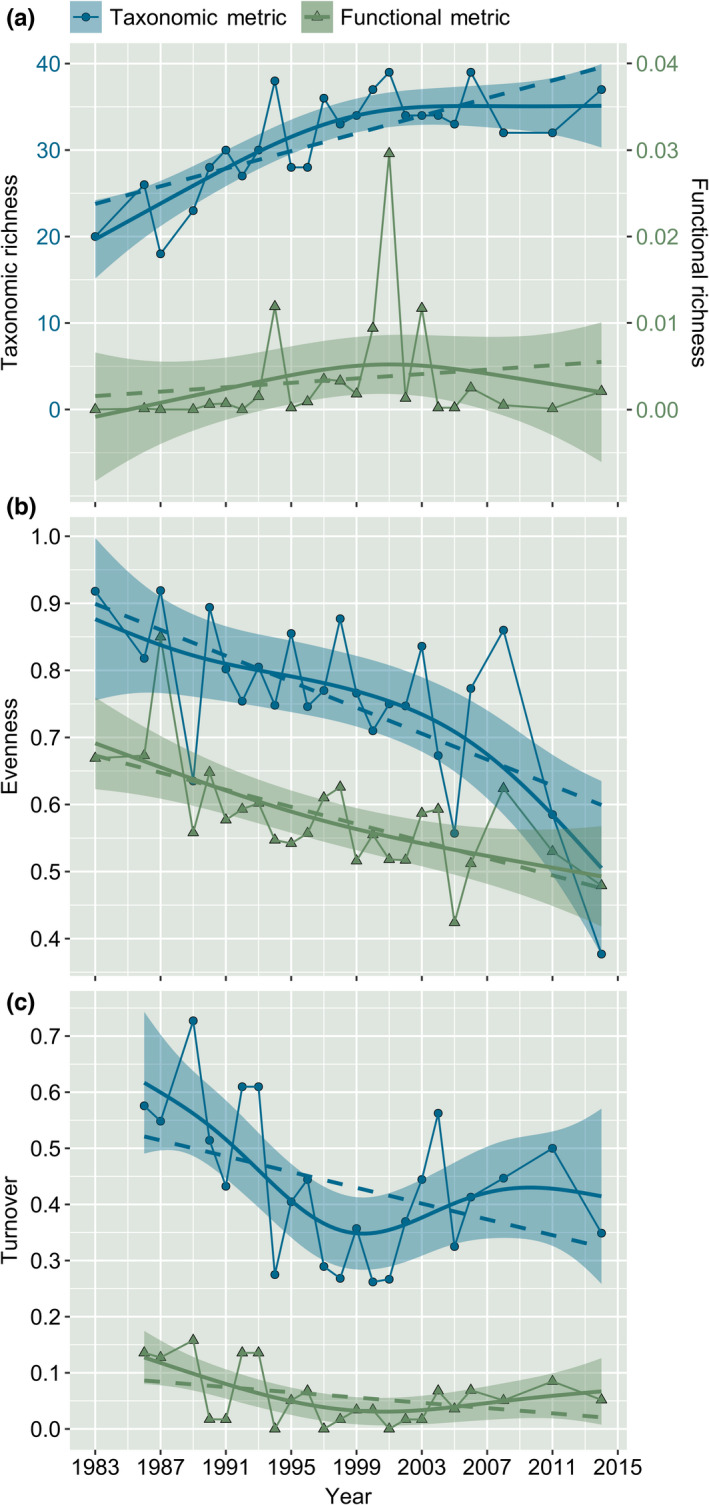
Comparison of trends in taxonomic and functional diversity. Dashed lines represent linear regression models (lm), whereas solid lines represent non‐linear regression models (gam, knots [*k*] = 6)

Trends of taxonomic and functional diversity (richness, evenness, turnover) were consistently analogous. However, the functional metric trends were lower compared with the taxonomic metrics. Taxonomic richness and FRic trends were collinear (*R* ^2^ = 0.845, *p* < .001; Figure 4a). Taxonomic richness increased significantly over the observation period (slope [S]: 5 ± 1 × 10^−1^, *p* < .001), whereas increases in FRic were not significant (*S*: 1 ± 1 × 10^−3^, *p* = .468; Table 2). Similarly, taxonomic evenness and FEve were collinear (*R* ^2^ = .722, *p* < .001; Figure 4b), with both significantly declining (taxonomic evenness S: −1 × 10^−2^ ± 3 × 10^−3^, *p* = .001; FEve *S*: −6 ± 2 × 10^−3^, *p* = .001; Table 2). Temporal taxonomic turnover (mean turnover = 0.435) declined over the observation period (*S*: −7 ± 3 × 10^−3^, *p* = .05; Figure 4c). Functional turnover (mean turnover = 0.058) was collinear to taxonomic turnover (*R* ^2^ = .78, *p* < .001), but did not significantly vary with time (*S*: −2 ± 1 × 10^−3^, *p* = .092). In all cases, non‐linear regression analysis supported the trends identified using linear regression analysis (Figure 4).

### 3.4 Functional redundancy

**FIGURE 5:**
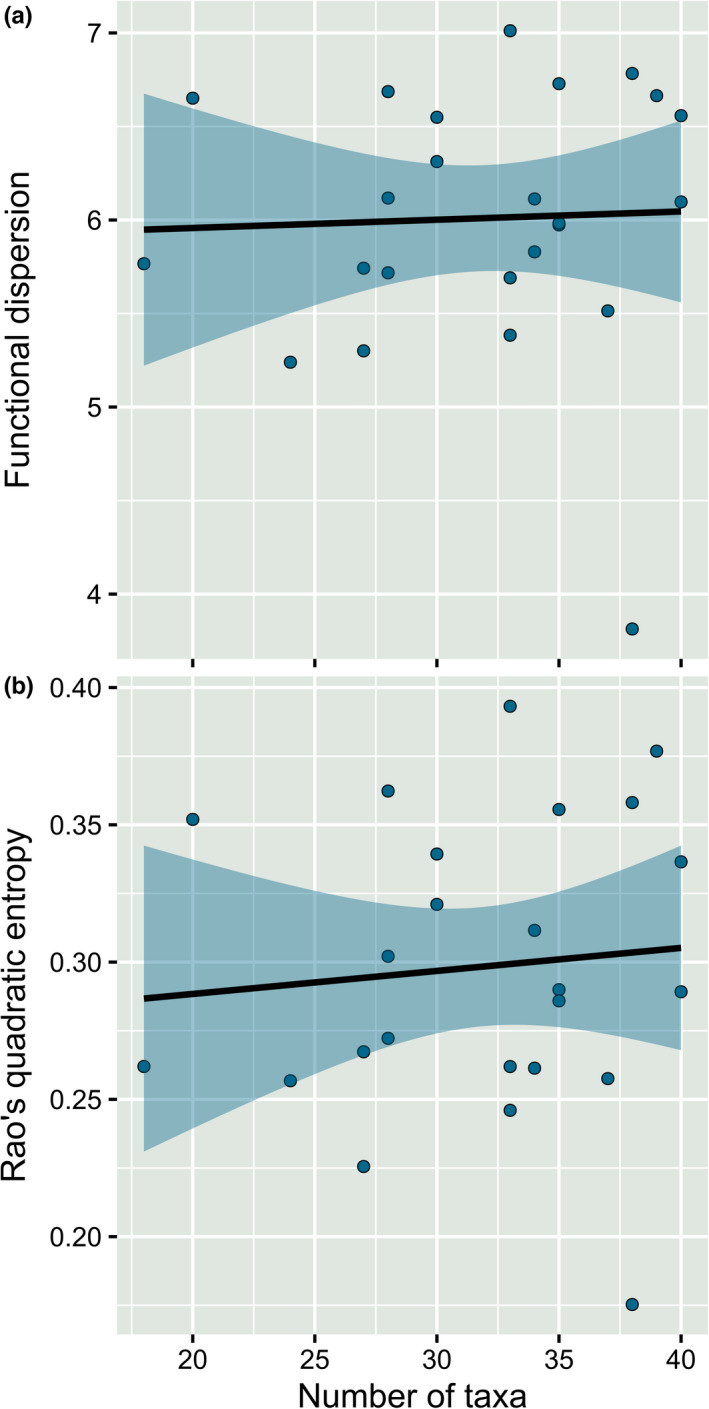
Linear regression models (lm) exploring the relationship between taxonomic diversity and functional diversity. (a) Functional dispersion–species richness. (b) Rao's quadratic entropy–species richness

Taxonomic richness (i.e., number of taxa) and functional diversity were not related (Figure 5). Linear regression analysis revealed shallow slopes for both functional diversity metrics: FDis (*S*: 4 × 10^−3^ ± 2.5 × 10^−2^, *R* ^2^ = −0.044, *p* = .860) and RaoQ (*S*: 2 ± 1 × 10^−3^, *R* ^2^ = −0.036, *p* = .662), indicating a high degree of functional redundancy within the macroinvertebrate communities of the Grosse Ohe River.

## 4 DISCUSSION

The recovery of stream macroinvertebrate taxonomic diversity (TD) was accompanied by increases in functional diversity (FD) over 32 years in a German national park. However, changes in FD were less pronounced and lagged behind taxonomic recovery. These results suggest the macroinvertebrate community contains an increasing degree of functional redundancy. Comparisons of both taxonomic and functional recovery processes in invertebrate communities are essential to deepen our understanding of the complexity of ecological processes such as recovery from anthropogenic stressors and can help guide conservation efforts.

### 4.1 Changes in functional diversity

In support of *H*_1a_, in which we predicted recovery of FD of the Grosse Ohe macroinvertebrate communities, functional richness (FRic) and functional divergence (FDiv) tended to increase, though not significantly. In contrast, functional evenness (FEve) declined. The occupied niche space of the communities also changed, increasing until ~2000, then decreasing, in partial support for *H*_1b_.

The non‐linear trend in FRic closely followed taxonomic recovery and restructuring patterns in Baker et al. (2021). In the first half of the observation period, FRic paralleled increased taxonomic recovery likely in response to acidification mitigation. However, after the year 2000, when both bark beetle outbreaks caused increasing nitrate levels and temperatures began noticeably rising (Baker et al., 2021), we observed declines in FRic and thus a reduction in the amount of occupied niche space. Decoupling of TD and FD is in alignment with a previous study investigating macroinvertebrate communities around natural large wood build‐ups in a British lowland river (Magliozzi et al., 2020).

The continuous decline in FEve over the observation period was driven by changes in taxonomic richness, as shown by the null model analysis (Appendix S3 Figure 1b). Such decline in FEve was quantitatively similar to the decline in taxonomic evenness and is indicative of an unequal distribution of taxa (some parts of the niche space are unoccupied, while others are densely populated; Mouchet et al., 2010). This could be due to the increasing unequal use of resources within the Grosse Ohe macroinvertebrate communities. Taxonomically, changes in FEve are likely linked to the increased dominance of the Diptera following nutrient disturbances and subsequent climate warming. The increased dominance of the Diptera has resulted in increases in certain trait combinations (smaller body sizes, multivoltine, passive aerial dispersers, resistant forms, and specialized breathing apparatuses such as a spiracle and temporarily or permanently attached apparatus; please refer to Appendix S5 regarding changes in traits through time). Considering the impacts of global climate change (IPPC, 2013), the Diptera may have an advantage over taxa with more unfavorable traits (large body sizes, aquatic dispersing, univoltine, lack of resistant forms, regiment respiring, crawlers). This is in agreement with results from Alpine catchments in France (Bruno et al., 2019). Bruno et al. (2019) found that resultant from climate change‐related stressors (flow reduction and rising water temperatures), specific trait combinations (smaller body sizes, multivoltine, aerial dispersers, etc.) became more common between the 1970s and 2010s, causing Alpine freshwater communities to resemble those of high‐order Mediterranean river systems. If such patterns hold for other low‐order mountainous streams similar to those for the Grosse Ohe, it is plausible that persistent, cold‐dwelling glacial relics (e.g., members of the Plecoptera and Trichoptera) may be further pressured as they are already limited by summit traps (Sauer et al., 2011). Moreover, if freshwater communities in protected, low‐order mountainous streams become more like those further down the river continuum (biotic homogenization; Haase et al., 2019; Olden, 2006), associated alterations to productivity, biogeochemistry, and nutrient cycles (Van Looy et al., 2016; Woodward et al., 2010) may induce additional changes in the future.

Of the many measures of the divergence of functional traits (FDiv; FDis; RaoQ; FDist), only FDiv showed a significant trend, increasing over time. This increase is indicative of a differentiation between taxa within the community, whereby the most abundant taxa (in this case, the Diptera) have a degree of functional dissimilarity to other taxa within the community (confirmed by our functional distinctiveness analysis) and therefore may not necessarily use the same resources as the rest of the community (Mouchet et al., 2010). This is particularly interesting because it may suggest that, in lieu of the findings that the Diptera are proportionally replacing other taxa within the community likely through competition (Baker et al., 2021), some Diptera might also be occupying other areas of the niche space. This could present an early indication that changing environmental conditions such as warming waters may already be indirectly altering fundamental ecosystem processes through their effects on biotic filtering.

Interestingly, functional dispersion (FDis) and Rao´s quadratic entropy (RaoQ) metrics, both measures of divergence and uncorrelated to taxonomic richness (Swenson, 2014), did not show significant change throughout the observation period. Although collinear, FDis and RaoQ can vary with regard to their interpretations: FDis is a measure of niche differentiation and can be used to judge the amount of functional redundancy within the community (Rosenfeld, 2002; Wellnitz & Poff, 2001), whereas RaoQ is a continuous measure of FD, which incorporates both elements of the evenness and richness dimensions (Weigelt et al., 2008). Due to its “*inclusive”* nature, RaoQ is one of the most widely used metrics in functional ecological studies (Schmera et al., 2017). However, considering the discrepancies (trend strength and direction) between the functional metrics used in the present study, we suggest increased explorations of all aspects of FD (i.e., richness, regularity, divergence). While FDis and RaoQ did not vary significantly with time, we do observe slight declines toward the latter part of our observation period. This decline may suggest that further functional changes may just be beginning, albeit at a gradual rate compared with that of taxonomic changes.

### 4.2 Functional distinctiveness

Although our distinctiveness analysis cannot discriminate between species‐specific competition within the niche space, the shifts in dominance between the Plecoptera, Trichoptera, and Diptera (Baker et al., 2021) suggest that the observed decline in FEve and increase in FDiv are likely resultant from an increase in both richness and abundance of the Diptera. Accordingly, we found heterogeneity between the FDist of different taxonomic groups in support of *H*_2_, with the Diptera and Coleoptera being functionally distinct from the other main biotic groups of Plecoptera and Trichoptera.

Due to their occurrence at high altitudes, low average temperatures, and position relative to the source (i.e., low‐order streams; Vannote et al., 1980), headwater streams such as the Grosse Ohe River are typically home to rare (Heino, 2005), specialist (Clarke et al., 2008), stenothermal taxa, such as Ephemeroptera, Plecoptera, and Trichoptera (Sauer et al., 2011). Changes in abiotic conditions of the Grosse Ohe River have been accompanied by increases in both abundance and richness of dipteran taxa, some of which are functionally similar to, and may be competing with, the Plecoptera and Trichoptera (Baker et al., 2021). However, through acidification mitigation, climate warming, and associated niche changes, more functionally distinct and recently established Diptera are possibly expanding their distribution, in turn occupying new, previously uninhabited parts of the niche space.

### 4.3 Comparison between taxonomic and functional changes: Functional redundancy

In agreement with both *H*_3a_ and *H*_3b_, taxonomic recovery of the Grosse Ohe communities over time has induced lesser, yet important changes in ecosystem functioning. The combined changes in TD and FD reveal a high degree of functional redundancy (supported by our null model analysis, which showed lower‐than‐expected functional richness toward the end of our observation period; Appendix S3, Figure 1a), suggesting that despite linear increases in TD over time, *newly* established taxa have functional traits similar to the already present species, while others may have introduced new functional traits into the community, in partial support for *H*_4_.

The introduction of both unique and similar traits by the new taxa may suggest a further replacement of the remaining, acid‐tolerant specialist species with that of more acid‐sensitive generalists. The replacement of specialists with generalists is a widely occurring phenomenon (Olden et al., 2018) and is likely resulting in more generalized functional characteristics (Haubrock et al., 2021). However, this replacement over the long term will lead to a loss of those unique traits carried by specialized species. Nevertheless, due to past acidification and continued biological recovery, it appears that the Grosse Ohe macroinvertebrate communities are more resilient than anticipated, a resilience likely linked with an increase in generalist species (see Appendix S5) and functional redundancy. While the high functional resilience of generalists seems advantageous, such generalist‐dominated communities can reflect a low level of specialization and increased biotic homogenization. A loss of taxonomic diversity (reduced percentage of specialist species) may lead to unforeseen impacts, for example, on the spatial insurance (see insurance hypothesis; Yachi & Loreau, 1999) of ecosystems and the continued conservation of biodiversity.

### 4.4 General conclusions and future outlooks

In a changing world, the rise of generalist species (and subsequent loss of specialists) appears to be a “blind” selection process (possibly resultant from natural reward; Gilbert, 2020) whereby natural systems maintain ecosystem functionality despite the continued loss or gain of species. Notwithstanding the “gloomy” reports of global entomofaunal communities (Wagner et al., 2021), there is growing support for general increases in freshwater biodiversity (Haase et al., 2019; Pilotto et al., 2020). However, long‐term taxonomic changes are not always easily interpreted and can sometimes be contradictory (Baranov et al., 2020). Here, we show that despite taxonomic gains of a community through time, the recovery of ecosystem functionality occurs at a more gradual pace, lagging behind that of taxonomic recovery. Exploring the rate of change between taxonomic and functional recovery and degradation remains an interesting avenue for future research. Shifts in functional diversity are likely more relevant toward informing conservation efforts than taxonomic diversity alone (Floury et al., 2018; Mouton et al., 2020; van Looy et al., 2016). Given our limited but increasing knowledge regarding functional traits, more autecological studies are needed to gain detailed trait information at the species level (Schmera et al., 2017). Furthermore, we recommend that future studies investigate which traits are the most at risk to ongoing anthropogenic stressors so that conservation efforts can be adapted and tailored to safeguard specific facets of diversity.

This study showcases the importance of long‐term studies when investigating complex ecological patterns and guiding conservation. These observed changes were prominent even in a protected area with limited effects of anthropogenic stressors. Biotic communities in ecosystems more severely impacted by anthropogenic activities are likely at greater risk of biodiversity loss and biotic homogenization. Accordingly, conservation efforts are caught between the protection of species or the preservation of ecosystem functioning. Therefore, more intense, harmonized monitoring efforts are needed (Haase et al., 2018), particularly in biodiverse areas, which can aid in gaining a deeper understanding of the restructuring of taxonomic and functional diversity in the Anthropocene.

## CONFLICTS OF INTEREST

The authors have no conflicts of interest.

## AUTHOR CONTRIBUTIONS

**Nathan Jay Baker:** Conceptualization (lead); Data curation (lead); Formal analysis (lead); Investigation (lead); Validation (equal); Visualization (lead); Writing‐original draft (lead); Writing‐review & editing (lead). **Francesca Pilotto:** Formal analysis (supporting); Investigation (supporting); Validation (equal); Writing‐review & editing (equal). **Phillip J. Haubrock:** Formal analysis (supporting); Validation (equal); Writing‐review & editing (equal). **Burkhard Beudert:** Data curation (supporting); Validation (equal); Writing‐review & editing (equal). **Peter Haase:** Conceptualization (supporting); Funding acquisition (lead); Investigation (supporting); Supervision (lead); Validation (equal); Writing‐review & editing (equal).

## Supporting information

Appendix S1

Appendix S2

Appendix S3

Appendix S4

Appendix S5

Video S1

## Data Availability

The complete taxon list, adjusted taxon list, and functional trait data are available on Dryad https://doi.org/10.5061/dryad.9s4mw6mht
