# Supplementary material for: Multidecadal changes in functional diversity lag behind the recovery of taxonomic diversity
Source: Ecol Evol. 2021 Nov 23;11(23):17471–84. doi: 10.1002/ece3.8381 (PMC8668763; doi:10.1002/ece3.8381)
Supplement: Supplementary file 1 — Appendix S1 [file ECE3-11-17471-s002.pdf]

Appendix 1: Quality control measures

1 Comparison of taxonomic metrics used in Baker et al. (2021)

Table 1.1 Summary of the Mann-Kendall trend analysis conducted on the various community metrics calculated from the complete (170 taxa; Baker et al. 2021) and taxonomically adjusted (70 taxa) taxa lists. P-values ≤ 0.05 are indicative of monotonic trends (in bold).

Taxa list	Metric	Z statistic	P-value	Mann-Kendall Tau	Sen's slope
	**Tabund**	**3.909**	**<0.001*** **	**0.589**	**42.867**
	**EPT_perc**	**−2.694**	**0.007** **	**−0.407**	**−1.729**
	Num_Taxa	1.917	0.055	0.299	0.460
**Complete**	Num_Genera	1.777	0.076	0.277	0.352
	Num_Fam	1.524	0.128	0.238	0.204
	**Margalef**	**3.197**	**<0.001*** **	**0.482**	**0.080**
	**Evenness**	**−2.048**	**0.041***	**−0.308**	**−0.004**
	**Tabund**	**3.845**	**<0.001*** **	**0.565**	**44.809**
	**EPT_perc**	**-2.580**	**0.010** **	**−0.380**	**−1.623**
	Num_Taxa	1.400	0.161	0.213	0.177
**Adjusted**	Num_Genera	1.400	0.161	0.213	0.197
	Num_Fam	1.268	0.205	0.194	0.171
	Margalef	1.414	0.157	0.210	0.034
	**Evenness**	**-2.654**	**0.008** **	**-0.391**	**-0.008**

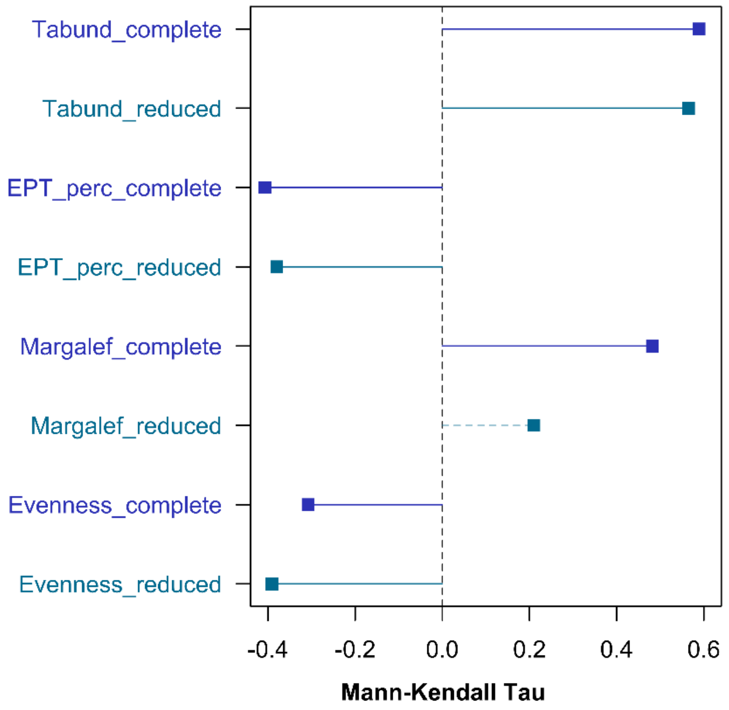

Figure 1.1 Mann-Kendall trend analysis indicating the similarities in trends (i.e., Mann-kendall Tau value) between selected taxonomic metrics used in Baker et al. (2021) based on the complete taxa list (170 taxa) and those in current manuscript using the taxonomically adjusted taxa list (70 taxa). Solid lines represent significant (p ≤ 0.05) monotonic trends, whereas dashed lines represent no significant (p > 0.05) trend.

2 Comparison of richness metrics

Table 2.1 Taxonomic and functional richness of the macroinvertebrate communities over time. 'Complete taxa list' refers to taxonomic richness (i.e., number of taxa) calculated using the taxa list from Baker et al. (2021) containing 170 taxa. 'Adjusted taxa list' refers to taxonomic richness calculated using the adjusted taxa list containing 70 taxa used for the coding of trait data. 'Functional dataset' refers to the calculated functional richness (FRic) of the community over time.

Year	Complete taxa list	Adjusted taxa list	Functional dataset
**1983**	20	20	<0.0000
**1986**	27	26	0.0001
**1987**	22	18	<0.0000
**1989**	18	23	<0.0000
**1990**	24	28	0.0006
**1991**	30	30	0.0007
**1992**	31	27	<0.0000
**1993**	32	30	0.0015
**1994**	35	38	0.0119
**1995**	29	28	0.0002
**1996**	26	28	0.0009
**1997**	40	36	0.0035
**1998**	31	33	0.0033
**1999**	29	34	0.0018
**2000**	38	37	0.0094
**2001**	38	39	0.0296
**2002**	32	34	0.0013
**2003**	36	34	0.0117
**2004**	40	34	0.0002
**2005**	37	33	0.0002
**2006**	51	39	0.0025
**2008**	36	32	0.0005
**2011**	43	32	0.0001
**2014**	44	37	0.0021

Table 2.2 Generalised additive model output reflecting the change in richness over time. p-values are approximate for the smooth term (Year). Values highlighted in bold represent significant smoothers and thus a significant non-linear change in a metric over time ($p \leq 0.001$; Zuur et al. 2009).

	Adjusted R^2	Explained deviance (%)	REML estimation	Smooth term (Year)			
				Estimated degrees of freedom	Reference degrees of freedom	F-statistic	p-value (approximate)
**Complete taxa list**	**0.669**	**68.3**	**67.911**	**1.000**	**1.000**	**47.48**	**<0.001*** **
**Adjusted taxa list**	**0.657**	**69.6**	**62.286**	**2.605**	**3.206**	**13.94**	**<0.001*** **
Functional dataset	0.063	13.9	-76.126	1.874	2.345	0.884	0.390

Table 2.3 Linear regression output reflecting the change in richness over time. p-values indicate a significant correlation between richness and time (Year). Values highlighted in bold represent significant trends ($p \leq 0.05$; Zuur et al. 2009).

	Degrees of freedom	Residual standard error	Multiple R^2	Adjusted R^2	Covariate (Year)			
					Estimate	Standard error	t-value	p-value
**Complete taxa list**	**22**	**4.588**	**0.683**	**0.669**	**0.816**	**0.118**	**6.891**	**<0.001*****
**Adjusted taxa list**	**22**	**4.004**	**0.525**	**0.503**	**0.510**	**0.103**	**4.931**	**<0.001*****
Functional dataset	22	0.007	0.024	-0.020	0.000	0.000	0.738	0.468

Table 2.4 Spearman's rank correlation highlighting the collinearity between richness calculated using the 'complete taxa list', 'adjusted taxa list' and 'functional dataset'. Values highlighted in bold represent a strong pairwise correlation between the variables ($p \leq 0.05$).

Variable 1	Variable 2	R^2	p-value
**Complete taxa list**	**Adjusted taxa list**	**0.813**	**<0.001*****
**Complete taxa list**	**Functional dataset**	**0.513**	**0.010****
**Adjusted taxa list**	**Functional dataset**	**0.845**	**<0.001*****

Table 2.5 Paired t-tests highlighting the differences in group means between richness calculated using the 'complete taxa list', ' adjusted taxa list' and 'functional dataset'. Cells highlighted in bold represent significant differences in group means between the variables ($p \leq 0.05$).

Variable 1	Variable 2	t-value	Mean of differences	p-value
Complete taxa list	Adjusted taxa list	1.769	1.625	0.090
**Complete taxa list**	**Functional dataset**	**20.199**	**32.872**	**<0.001*****
**Adjusted taxa list**	**Functional dataset**	**26.958**	**31.247**	**<0.001*****

3 Comparison of richness metrics

Table 3.1 Taxonomic and functional evenness of the macroinvertebrate communities over time. 'Complete taxa list' refers to taxonomic evenness calculated using the taxa list from Baker et al. (2021) containing 170 taxa. 'Adjusted taxa list' refers to taxonomic evenness calculated using the adjusted taxa list containing 70 taxa used for the coding of trait data. 'Functional dataset' refers to the calculated functional evenness (FEve) of the community over time.

Year	Complete taxa list	Adjusted taxa list	Functional dataset
**1983**	0.93	0.918	0.669
**1986**	0.84	0.818	0.673
**1987**	0.93	0.919	0.85
**1989**	0.6	0.635	0.558
**1990**	0.92	0.894	0.648
**1991**	0.81	0.802	0.577
**1992**	0.78	0.754	0.593
**1993**	0.86	0.805	0.602

Table 3.1 Continued.

**1994**	0.79	0.748	0.547
**1995**	0.88	0.855	0.542
**1996**	0.77	0.746	0.557
**1997**	0.85	0.77	0.61
**1998**	0.89	0.877	0.626
**1999**	0.8	0.766	0.516
**2000**	0.79	0.71	0.555
**2001**	0.78	0.75	0.518
**2002**	0.78	0.747	0.517
**2003**	0.87	0.836	0.587
**2004**	0.75	0.673	0.593
**2005**	0.56	0.557	0.424
**2006**	0.78	0.773	0.512
**2008**	0.88	0.86	0.624
**2011**	0.78	0.585	0.53
**2014**	0.65	0.377	0.479

Table 3.2 Generalised additive model output reflecting the change in evenness over time. p-values are approximate for the smooth term (Year). Values highlighted in bold represent significant smoothers and thus a significant non-linear change in a metric over time ($p \leq 0.001$; Zuur et al. 2009).

				Smooth term (Year)			
	Adjusted R^2	Explained deviance (%)	REML estimation	Estimated degrees of freedom	Reference degrees of freedom	F-statistic	p-value (approximate)
Complete taxa list	0.131	16.9	-18.949	1.000	1.000	4.466	0.046*
Adjusted taxa list	0.456	51.3	-16.982	2.402	2.975	6.617	0.002**
Functional dataset	0.386	42.5	-25.527	1.479	1.818	7.446	0.003**

Table 3.3 Linear regression output reflecting the change in evenness over time. p-values indicate a significant correlation between temporal turnover and time (Year). Values highlighted in bold represent significant trends ($p \leq 0.05$; Zuur et al. 2009).

					Covariate (Year)			
	Degrees of freedom	Residual standard error	Multiple R^2	Adjusted R^2	Estimate	Standard error	t-value	p-value
**Complete taxa list**	**22**	**0.089**	**0.169**	**0.131**	**-0.005**	**0.002**	**-2.113**	**0.046***
**Adjusted taxa list**	**22**	**0.099**	**0.393**	**0.3651**	**-0.010**	**0.003**	**-3.771**	**0.001****
**Functional dataset**	**22**	**0.066**	**0.393**	**0.3653**	**-0.006**	**0.002**	**-3.774**	**0.001****

Table 3.4 Spearman's rank correlation highlighting the collinearity between evenness calculated using the 'complete taxa list', 'adjusted taxa list' and 'functional dataset'. Values highlighted in bold represent a strong pairwise correlation between the variables ($p \leq 0.05$).

Variable 1	Variable 2	R^2	p-value
**Complete taxa list**	**Adjusted taxa list**	**0.943**	**<0.001*****
**Complete taxa list**	**Functional dataset**	**0.733**	**<0.001*****
**Adjusted taxa list**	**Functional dataset**	**0.722**	**<0.001*****

Table 3.5 Paired t-test highlighting the differences in group means between evenness calculated using the 'complete taxa list', 'adjusted taxa list' and 'functional dataset'. Cells highlighted in bold represent significant differences in group means between the variables (p ≤ 0.05).

Variable 1	Variable 2	t-value	Mean of differences	p-value
**Complete taxa list**	**Adjusted taxa list**	**3.374**	**0.044**	**0.003****
**Complete taxa list**	**Functional dataset**	**16.094**	**0.223**	**<0.001*****
**Adjusted taxa list**	**Functional dataset**	**9.638**	**0.179**	**<0.001*****

4 Comparison of turnover metrics

Table 4.1 Taxonomic and functional turnover of the macroinvertebrate communities over time. ‘Complete taxa list’ refers to taxonomic turnover calculated using the taxa list from Baker et al. (2021) containing 170 taxa. ‘Adjusted taxa list’ refers to taxonomic turnover calculated using the adjusted taxa list containing 70 taxa used for the coding of trait data. ‘Functional dataset’ refers to the turnover of traits and therefore trait membership states within the community over time.

Year	Complete taxa list			Adjusted taxa list			Functional dataset		
	Total	Appearance	Disappearance	Total	Appearance	Disappearance	Total	Appearance	Disappearance
**1983**	-	-	-	-	-	-	-	-	-
**1986**	0.800	0.576	0.136	0.576	0.394	0.182	0.136	0.068	0.068
**1987**	0.649	0.548	0.127	0.548	0.129	0.419	0.127	0.000	0.127
**1989**	0.838	0.727	0.158	0.727	0.455	0.273	0.158	0.158	0.000
**1990**	0.825	0.514	0.017	0.514	0.314	0.200	0.017	0.017	0.000
**1991**	0.667	0.432	0.017	0.432	0.243	0.189	0.017	0.017	0.000
**1992**	0.745	0.610	0.136	0.610	0.268	0.341	0.136	0.000	0.136
**1993**	0.714	0.610	0.136	0.610	0.341	0.268	0.136	0.136	0.000
**1994**	0.611	0.275	0.000	0.275	0.250	0.025	0.000	0.000	0.000
**1995**	0.660	0.405	0.051	0.405	0.071	0.333	0.051	0.000	0.051
**1996**	0.705	0.444	0.068	0.444	0.222	0.222	0.068	0.051	0.017
**1997**	0.500	0.289	0.000	0.289	0.263	0.026	0.000	0.000	0.000
**1998**	0.569	0.268	0.017	0.268	0.098	0.171	0.017	0.017	0.000
**1999**	0.548	0.357	0.034	0.357	0.190	0.167	0.034	0.000	0.034
**2000**	0.642	0.262	0.034	0.262	0.167	0.095	0.034	0.034	0.000
**2001**	0.545	0.267	0.000	0.267	0.156	0.111	0.000	0.000	0.000
**2002**	0.489	0.370	0.017	0.370	0.130	0.239	0.017	0.000	0.017
**2003**	0.709	0.444	0.017	0.444	0.222	0.222	0.017	0.017	0.000
**2004**	0.758	0.563	0.068	0.563	0.271	0.292	0.068	0.000	0.068
**2005**	0.566	0.325	0.036	0.325	0.150	0.175	0.036	0.018	0.018
**2006**	0.508	0.413	0.069	0.413	0.283	0.130	0.069	0.052	0.017
**2008**	0.627	0.447	0.051	0.447	0.149	0.298	0.051	0.034	0.017
**2011**	0.593	0.500	0.085	0.500	0.250	0.250	0.085	0.017	0.068
**2014**	0.532	0.349	0.052	0.349	0.233	0.116	0.052	0.052	0.000
**Ave.**	0.643	0.435	0.058	0.435	0.228	0.206	0.058	0.030	0.028

Table 4.2 Generalised additive model output reflecting the change in temporal turnover over time. p-values are approximate for the smooth term (Year). Values highlighted in bold represent significant smoothers and thus a significant non-linear change in a metric over time ($p \leq 0.001$; Zuur et al. 2009).

	Adjusted R^2	Explained deviance (%)	REML estimation	Smooth term (Year)			
				Estimated degrees of freedom	Reference degrees of freedom	F-statistic	p-value (approximate)
Complete taxa list	0.358	40.7	-18.617	1.698	2.115	6.239	0.007**
Adjusted taxa list	0.423	50.5	-13.535	3.099	3.738	4.246	0.012*
Functional dataset	0.338	41.3	-33.167	2.493	3.07	3.925	0.023*

Table 4.3 Linear regression output reflecting the change in functional turnover through time. p-values indicate a significant correlation between temporal turnover and time (Year). Values highlighted in bold represent significant trends ($p \leq 0.05$; Zuur et al. 2009).

	Degrees of freedom	Residual standard error	Multiple R^2	Adjusted R^2	Covariate (Year)			
					Estimate	Standard error	t-value	p-value
**Complete taxa list**	**21**	**0.087**	**0.349**	**0.318**	**-0.008**	**0.002**	**-3.354**	**0.003****
**Adjusted taxa list**	**21**	**0.121**	**0.171**	**0.131**	**-0.007**	**0.003**	**-2.079**	**0.050***
Functional dataset	21	0.047	0.129	0.088	-0.002	0.001	-1.764	0.092

Table 4.4 Spearman's rank correlation highlighting the collinearity between turnover calculated using the 'complete taxa list', 'adjusted taxa list' and 'functional dataset'. Values highlighted in bold represent a strong pairwise correlation between the variables ($p \leq 0.05$).

Variable 1	Variable 2	R^2	p-value
**Complete taxa list**	**Adjusted taxa list**	**0.740**	**<0.001*****
**Complete taxa list**	**Functional dataset**	**0.500**	**0.015***
**Adjusted taxa list**	**Functional dataset**	**0.780**	**<0.001*****

Table 4.5 Paired t-test highlighting the differences in group means between turnover calculated using the 'complete taxa list', 'adjusted taxa list' and 'functional dataset'. Cells highlighted in bold represent significant differences in group means between the variables ($p \leq 0.05$).

Variable 1	Variable 2	t-value	Mean of differences	p-value
**Complete taxa list**	**Adjusted taxa list**	**12.021**	**0.209**	**<0.001*****
**Complete taxa list**	**Functional dataset**	**32.008**	**0.586**	**<0.001*****
**Adjusted taxa list**	**Functional dataset**	**19.808**	**0.377**	**<0.001*****

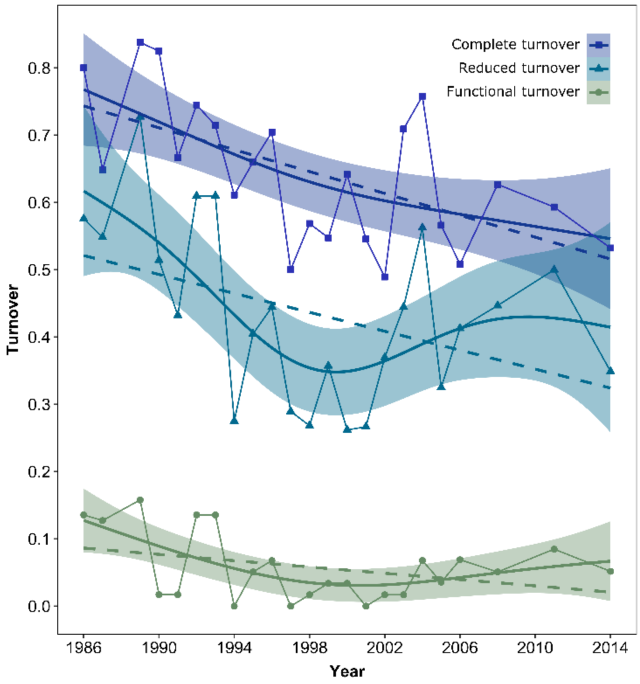

Figure 4.1 Comparison of turnover trends in the complete taxa list (purple), adjusted taxa list (blue) and functional dataset (green). Solid lines represent generalised additive models, whereas dashed lines represent linear regression.
