# Supplementary material for: Multidecadal changes in functional diversity lag behind the recovery of taxonomic diversity
Source: Ecol Evol. 2021 Nov 23;11(23):17471–84. doi: 10.1002/ece3.8381 (PMC8668763; doi:10.1002/ece3.8381)
Supplement: Supplementary file 2 — Appendix S2 [file ECE3-11-17471-s004.pdf]

Appendix 2: Trait groups and traits (i.e., modalities)

Table 1 Trait groups and traits (i.e., modalities) used for the calculation of functional metrics. Trait nomenclature follows Schmera et al. (2015).

Trait group	Unit	Trait	Short name	Trait type	Trait grouping feature	Database used
Maximal body size	mm	≤ 2.5; >2.5–5; >5–10;>10–20;>20–40;>40–80; >80	size1; size2; size3; size4; size5; size6; size7	Biological	Life cycle descriptors	Tachet 2010; STAR; DIPSERSE; www.freshwaterecology.info
Life cycle duration	year	≤ 1;> 1	life1; life2	Biological	Life cycle descriptors	Tachet 2010; STAR; DISPERSE; www.freshwaterecology.info
Potential number of cycles per year	–	< 1; 1; > 1	cycl1; cycl2; cycl3	Biological	Life cycle descriptors	Tachet 2010; STAR; www.freshwaterecology.info
Aquatic developmental stages	–	egg; larva; nymph; adult	aqua1; aqua2; aqua3; aqua4	Biological	Life cycle descriptors	Tachet 2010; STAR; www.freshwaterecology.info
Type of reproduction/ reproductive technique	–	ovoviviparity; isolated eggs, free (not fixed); isolated eggs, cemented; clutches, cemented or fixed; clutches, free; eggs or clutches in vegetation (endophytic); clutches, terrestrial; asexual reproduction	repr1; repr2; repr3; repr4; repr5; repr6; repr7; repr8	Biological	Behavioural aspects of reproduction or nutrition	Tachet 2010; STAR; www.freshwaterecology.info
Dispersal	–	aquatic passive; aquatic active; aerial passive; aerial active	disp1; disp2; disp3; disp4	Biological	Resistance/resilience potential	Tachet 2010; STAR; www.freshwaterecology.info
Resistance forms	–	eggs, statoblasts, gemmules; cocoons; using refuges to resist desiccation during droughts; diapause or dormancy; none	resi1; resi2; resi3; resi4; resi5	Biological	Resistance/resilience potential	Tachet 2010; STAR; www.freshwaterecology.info
Type of respiration/ respiration technique	–	tegument; gill; plastron; spiracle; hydrostatic vesicle	resp1; resp2; resp3; resp4; resp5	Biological	Physiological strategy	Tachet 2010; STAR; www.freshwaterecology.info
Substrate relation and locomotion	–	flier; surface swimmer; swimmer; crawler; burrower; interstitial; temporarily attached; almost permanently attached	loco1; loco2; loco3; loco4; loco5; loco6; loco7; loco8	Biological	Resistance/resilience potential	Tachet 2010; STAR; www.freshwaterecology.info
Type of food	–	fine sediment and microorganisms; detritus < 1mm; plant detritus ≥ 1mm; living microphytes; living macrophytes; dead animals; living microinvertebrates; living macroinvertebrates; vertebrates	food1; food2; food3; food4; food5; food6; food7; food8; food9	Biological	Behavioural aspects of reproduction or nutrition	Tachet 2010; STAR; www.freshwaterecology.info
Feeding habits	–	deposit feeder; shredder; scraper; filter feeder; piercer (plants or animals); predator (carver/engulfer/ swallower); predator; parasite	fhab1; fhab2; fhab3; fhab4; fhab5; fhab6; fhab7; fhab8	Biological	Behavioural aspects of reproduction or nutrition	Tachet 2010; STAR; www.freshwaterecology.info

References

AQEM Consortium. (2004). AQEMdip: AQEM data input program (STAR Database). Retrieved from http://www.eu-star.at

Sarremejane, R., Cid, N., Stubbington, R., Datry, T., Alp, M., Cañedo-Argüelles, M., … Bonada, N. (2020). DISPERSE, a trait database to assess the dispersal potential of European aquatic macroinvertebrates. Scientific Data, 7, 386. doi: 10.1038/s41597-020-00732-7

Schmera, D., Podani, J., Heino, J., Erős, T., & Poff, N. L. (2015). A proposed unified terminology of species traits in stream ecology. *Freshwater Science*, *34*, 823–830. doi: 10.1086/681623

Schmidt-Kloiber, A., & Hering, D. (2015). www.freshwaterecology.info – An online tool that unifies, standardises and codifies more than 20,000 European freshwater organisms and their ecological preferences. Ecological Indicators, 53, 271–282. doi: 10.1016/j.ecolind.2015.02.007

Tachet, H., Richoux, P., Bournaud, M., & Usseglio-Polatera, P. (2010). Invertébrés d’eau Douce: Systematique, Biologie, Ecologie. Paris: CNRS EDITIONS.
