# Supplementary material for: Multidecadal changes in functional diversity lag behind the recovery of taxonomic diversity
Source: Ecol Evol. 2021 Nov 23;11(23):17471–84. doi: 10.1002/ece3.8381 (PMC8668763; doi:10.1002/ece3.8381)
Supplement: Supplementary file 3 — Appendix S3 [file ECE3-11-17471-s001.pdf]

**Appendix 3: Null models**

## 1 Null model design

The measures of functional diversity described by Villéger et al. (2008), particularly functional richness (FRic) and evenness (FEve), have been found to be strongly positively correlated to taxonomic richness (Gotelli and Graves, 1996). Alternatively, other measures such as functional dispersion (FDis) and Rao's quadratic entropy (RaoQ) are, by design, uncorrelated to taxonomic richness (Swenson, 2014). Thus, to ensure that our conclusions based on the trends of FRic, FEve, and FDiv (Villéger et al., 2008) are robust, we used a null approach to correct for annual differences in taxonomic richness. Using a 'name shuffling' null model which randomizes the taxa names assigned to the traits combinations of the original trait dataset (Swenson, 2014), we generated a null distribution through 999 permutations. We then measured the standardised effect size (S.E.S.) of each functional metric as $(FD_{obs} - mean(FD_{null}))/sd(FD_{null})$, with $FD_{obs}$ being the observed functional diversity metric value and $FD_{null}$ being null distribution of the functional diversity metric based on 999 permutations (Kuczynski et al., 2018). A positive S.E.S value indicates that, for a given annual taxonomic richness, the observed functional diversity metric is higher than expected. A negative S.E.S. indicates that the observed functional diversity metric is lower than expected given the taxonomic richness in that year. Then, to determine the extent to which differences in taxonomic richness influenced the observed functional diversity metrics, we used a Pearson rank correlation to compare the unstandardized functional metrics (FRic, FEve, FDiv) with the standardized functional metrics (FRic.SES, FEve.SES, FDiv.SES).

## 2 Null model results

In general, trends in the S.E.S. functional metrics (Fig. 1, Table 1) paralleled those of the observed functional metrics (Fig. 1 in the main text). Thus, to limit metric redundancy, we chose only to present the results of the observed functional metrics in the main text.

Our correlation analysis revealed strong positive correlations between the observed functional metrics (in the main text) and S.E.S. functional metrics (FRic $R^2 = 0.83$, $p < 0.001$; FEve $R^2 = 0.57$, $p = 0.004$; FDiv $R^2 = 0.8$, $p < 0.001$). Moreover, the results of the null models

demonstrate that the changes through time in FRic and FEve (shown in the main text) are driven by the changes in taxonomic richness as here the trends in FRic.SES and FEve.SES are not significant (Table 2). Further, and although not significant, the FRic.SES trends confirm our results of high functional redundancy towards the end of the observation period as the functional richness was lower than expected given the taxonomic richness in those years. The increase in FDiv (shown in the main text) is paralleled by increases in FDiv.SES, showing low levels of divergence in the beginning of the study period and increases from the end of the 1990s.

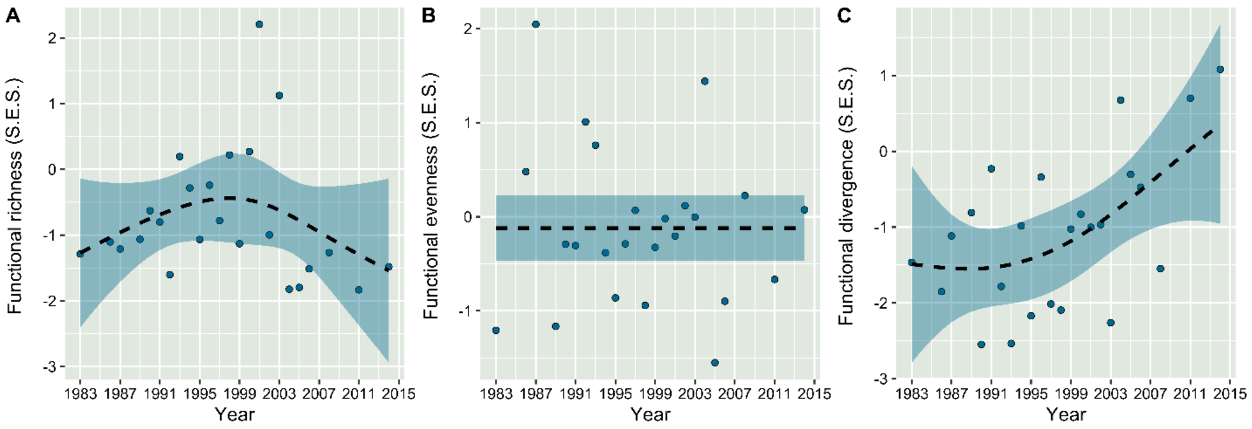


**Figure 1** Generalised additive models (gam) exploring the trends of the S.E.S. functional metrics through time (Year). The number of knots has been arbitrarily set at 6. Dashed lines represent no significant change over time (p ≥ 0.001; Zuur et al. 2009). **A**, S.E.S. functional richness (FRic.SES). **B**, S.E.S. functional evenness (FEve.SES). **C**, S.E.S. functional divergence (FDiv.SES).

**Table 1** Generalised additive model outputs reflecting the change in standardised functional metrics over time. Standardised functional metrics are represented as standardised effect sizes (S.E.S.) comparing observed values to expected values through a null model. Values highlighted in bold represent significant smoothers and thus a significant non-linear change in the standardized metric over time (p ≤ 0.001; Zuur et al. 2009).

| | | | | Smooth term (Year) | | | |
|---|---|---|---|---|---|---|---|
| | **Adjusted $R^2$** | **Explained deviance (%)** | **REML estimation** | **Estimated degrees of freedom** | **Reference degrees of freedom** | **F-statistic** | **p-value (approximate)** |
| FRic.SES | 0.16 | 21.7 | 33.402 | 1.643 | 5.000 | 0.858 | 0.088 |
| FEve.SES | 0.00 | 0.00 | 30.378 | 0.000 | 5.000 | 0.000 | 0.716 |
| FDiv.SES | 0.33 | 38.4 | 31.663 | 1.723 | 5.000 | 2.307 | 0.004** |

## 3 References


Gotelli, N. J., & Graves, G. R. (1996). Null models in ecology. Washington, D.C.: Smithsonian Institution Press.

Kuczynski, L., Côte, J., Toussaint, A., Brosse, S., Buisson, L., & Grenouillet, G. (2018). Spatial mismatch in morphological, ecological and phylogenetic diversity, in historical and contemporary European freshwater fish faunas. Ecography, 41(10), 1665–1674. doi: 10.1111/ecog.03611

Swenson, N. G. (2014). Functional and phylogenetic ecology in R. New York: Springer. doi: 10.1007/978-1-4614-9542-0

Villéger, S., Mason, N. W. H., & Mouillot, D. (2008). New multidimensional functional diversity indices for a multifaceted framework in functional ecology. Ecology, 89(8), 2290–2301. doi: 10.1890/07-1206.1

Zuur, A. F., Ieno, E. N., Walker, J. N., Saveliev, A. A., & Smith, G. M. (2009). Mixed effects models and extensions in ecology with R. New York: Springer. doi: 10.1017/CBO9781107415324.004
