# Supplementary material for: Multidecadal changes in functional diversity lag behind the recovery of taxonomic diversity
Source: Ecol Evol. 2021 Nov 23;11(23):17471–84. doi: 10.1002/ece3.8381 (PMC8668763; doi:10.1002/ece3.8381)
Supplement: Supplementary file 4 — Appendix S4 [file ECE3-11-17471-s006.pdf]

Appendix 4: Functional diversity metrics

Table 1 Annual functional diversity of the Grosse Ohe macroinvertebrate communities represented by distance-based functional metrics. Functional metrics were calculated using 11 trait groups with 63 traits (i.e., modalities; sensu Schmera et al., 2015). Due to the high number of traits used, PCoA dimensionality was reduced leading to a trait space comprised of the first 13 axes only; the quality of the reduced trait space was 0.754.

Year	Number of species per community	Number of functionally singular species	Functional Richness	Functional Evenness	Functional Divergence	Functional Dispersion	Rao's Quadratic Entropy	Functional distinctiveness
			FRic	FEve	FDiv	FDis	RaoQ	FDist
**1983**	20	18	0.0000	0.669	0.758	6.651	0.352	0.178
**1986**	27	26	0.0001	0.673	0.697	5.742	0.267	0.182
**1987**	18	18	0.0000	0.850	0.754	5.766	0.262	0.187
**1989**	24	23	0.0000	0.558	0.715	5.240	0.257	0.204
**1990**	28	28	0.0006	0.648	0.705	6.686	0.362	0.199
**1991**	30	30	0.0007	0.577	0.779	6.312	0.321	0.189
**1992**	27	27	0.0000	0.593	0.689	5.300	0.226	0.178
**1993**	30	29	0.0015	0.602	0.710	6.549	0.339	0.177
**1994**	39	39	0.0119	0.547	0.745	6.665	0.377	0.172
**1995**	28	27	0.0002	0.542	0.699	6.117	0.302	0.179
**1996**	28	28	0.0009	0.557	0.734	5.718	0.272	0.192
**1997**	37	36	0.0035	0.610	0.631	5.514	0.258	0.176
**1998**	34	33	0.0033	0.626	0.674	6.113	0.312	0.188
**1999**	35	33	0.0018	0.516	0.691	5.974	0.290	0.180
**2000**	38	36	0.0094	0.555	0.786	6.783	0.358	0.150
**2001**	40	40	0.0296	0.518	0.735	6.558	0.337	0.170
**2002**	35	33	0.0013	0.517	0.699	5.983	0.286	0.187
**2003**	35	33	0.0117	0.587	0.709	6.729	0.356	0.186
**2004**	34	32	0.0002	0.593	0.813	5.830	0.261	0.198
**2005**	33	31	0.0002	0.424	0.918	5.691	0.262	0.105
**2006**	40	38	0.0025	0.512	0.781	6.097	0.289	0.176
**2008**	33	31	0.0005	0.624	0.767	7.012	0.393	0.188
**2011**	33	31	0.0001	0.530	0.898	5.384	0.246	0.202
**2014**	38	36	0.0021	0.479	0.966	3.813	0.175	0.218

Table 2 Generalised additive model outputs reflecting the change in functional metrics over time. p-values are approximate for the smooth term (Year). Values highlighted in bold represent significant smoothers and thus a significant non-linear change in a metric over time (p ≤ 0.001; Zuur et al. 2009).

	Adjusted R^2	Explained deviance (%)	REML estimation	Smooth term (Year)			
				Estimated degrees of freedom	Reference degrees of freedom	F-statistic	p-value (approximate)
**log(FRic)**	**0.55**	**60.5**	**36.159**	**2.649**	**5**	**5.703**	**<0.001*****
**FEve**	**0.38**	**41.6**	**-26.956**	**1.351**	**5**	**2.819**	**<0.001*****
**FDiv**	**0.60**	**64.6**	**-31.172**	**2.559**	**5**	**6.956**	**<0.001*****
FDis	0.35	44.2	23.998	3.250	3.9	3.155	0.033*
RaoQ	0.09	16.8	-30.415	1.996	2.516	1.400	0.346
FDist	0.14	23.8	-50.610	2.547	3.141	1.494	0.237

Table 3 Linear regression output reflecting functional redundancy as the relationship between functional diversity (FDis and RaoQ) and taxonomic diversity (number of taxa). Trends are considered significant when $p \leq 0.05$ (Zuur et al. 2009).

	Degrees of freedom	Residual standard error	Multiple R^2	Adjusted R^2	Covariate (number of taxa)			
					Estimate	Standard error	t-value	p-value
**FDis**	22	0.708	0.002	−0.043	0.004	0.025	0.179	0.860
**RaoQ**	22	0.054	0.009	−0.036	0.001	0.002	0.443	0.662

References

Schmera, D., Podani, J., Heino, J., Erős, T., & Poff, N. L. (2015). A proposed unified terminology of species traits in stream ecology. Freshwater Science, 34(3), 823–830. doi: 10.1086/681623
