# Supplementary material for: Multidecadal changes in functional diversity lag behind the recovery of taxonomic diversity
Source: Ecol Evol. 2021 Nov 23;11(23):17471–84. doi: 10.1002/ece3.8381 (PMC8668763; doi:10.1002/ece3.8381)
Supplement: Supplementary file 5 — Appendix S5 [file ECE3-11-17471-s003.pdf]

Appendix 5: Change in traits through time.

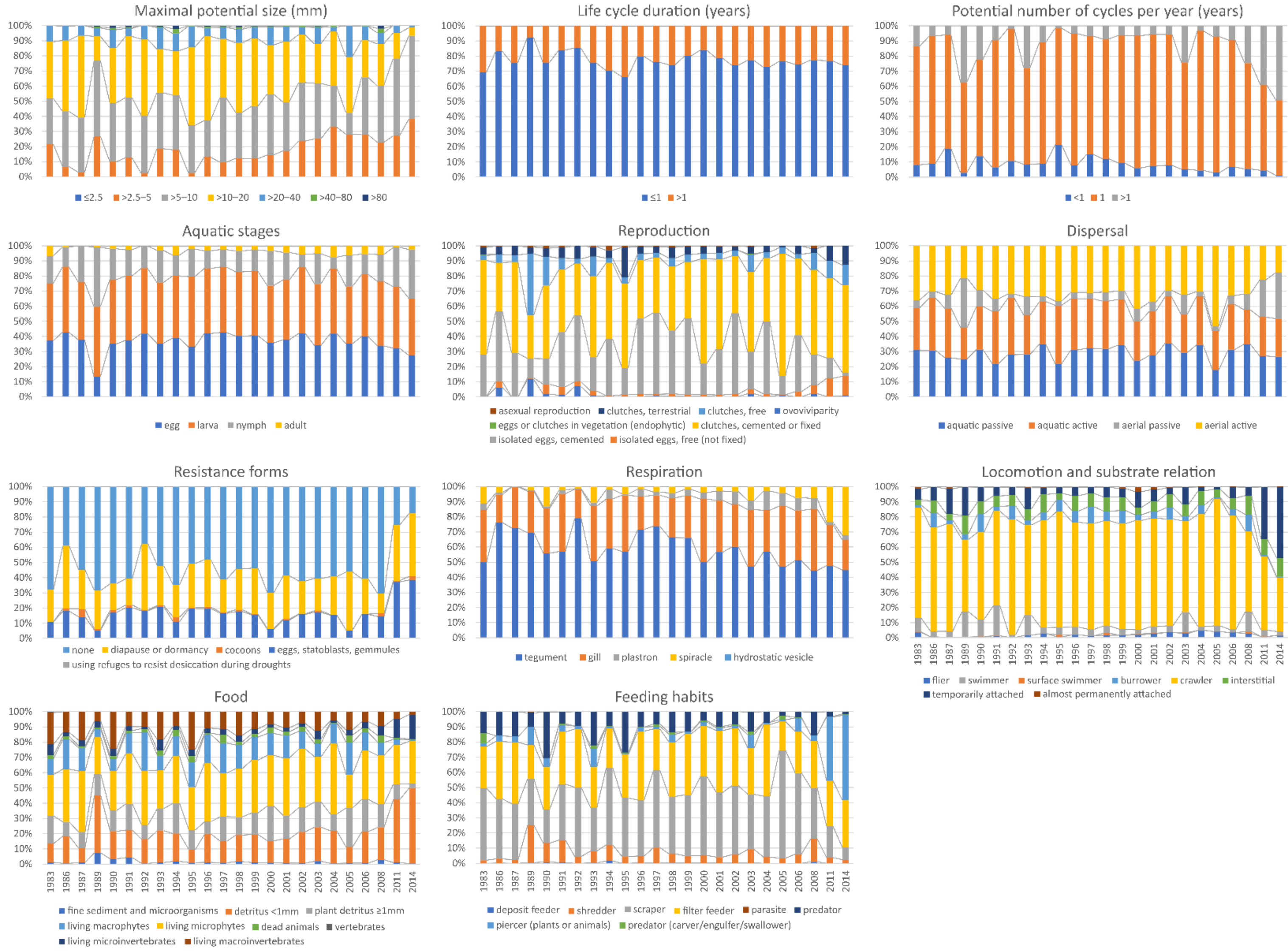

Figure 1 Stacked bar graph indicating the change in traits (i.e., modalities) through time. Y-axes represent the proportional occurrence of each trait.

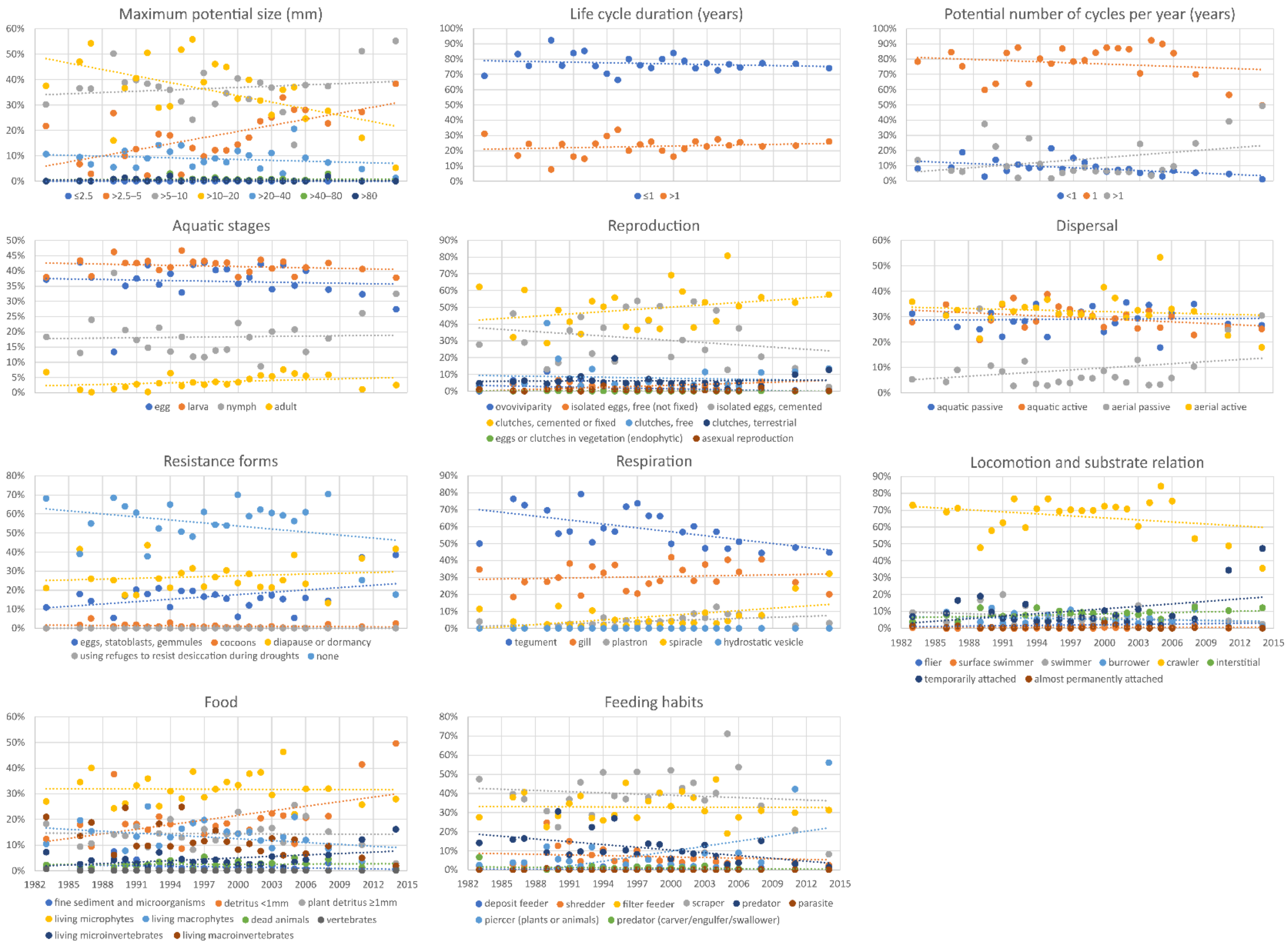

Figure 2 Regression lines indicating the change in traits (i.e., modalities) through time. Y-axes represent the proportional occurrence of each trait.
