# Supplementary figures and images for: Multidecadal changes in functional diversity lag behind the recovery of taxonomic diversity
Source: Ecol Evol. 2021 Nov 23;11(23):17471–84. doi: 10.1002/ece3.8381 (PMC8668763; doi:10.1002/ece3.8381)

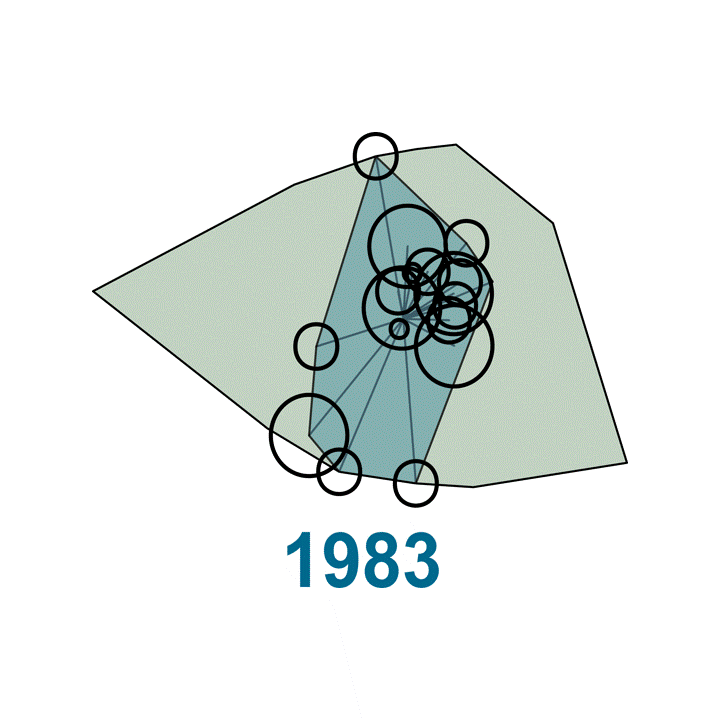

Supplement: Supplementary file 6 — Video S1 [file ECE3-11-17471-s005.gif]
